# Does implicit motor learning lead to greater automatization of motor skills compared to explicit motor learning? A systematic review

**DOI:** 10.1371/journal.pone.0203591

**Published:** 2018-09-05

**Authors:** Elmar Kal, Rens Prosée, Marinus Winters, John van der Kamp

**Affiliations:** 1 Department of Research & Development, Heliomare Rehabilitation Centre, Wijk aan Zee, The Netherlands; 2 Department of Human Movement Sciences, Faculty of Behavioural and Movement Sciences, VU University Amsterdam, Amsterdam Movement Sciences, Amsterdam, Institute of Brain and Behaviour Amsterdam, The Netherlands; 3 Department of Clinical Neuropsychology, Faculty of Behavioural and Movement Sciences, VU University Amsterdam, The Netherlands; 4 Research Unit of General Practice in Aalborg, Department of Clinical Medicine, Aalborg University, Aalborg, Denmark; 5 Research Centre for Exercise, School and Sport, Windesheim University of Applied Sciences, Zwolle, The Netherlands; Universidad de Granada, SPAIN

## Abstract

**Background:**

Implicit motor learning is considered to be particularly effective for learning sports-related motor skills. It should foster movement automaticity and thereby facilitate performance in multitasking and high-pressure environments. To scrutinize this hypothesis, we systematically reviewed all studies that compared the degree of automatization achieved (as indicated by dual-task performance) after implicit compared to explicit interventions for sports-related motor tasks.

**Methods:**

For this systematic review (CRD42016038249) conventional (MEDLINE, CENTRAL, Embase, PsycINFO, SportDiscus, Web of Science) and grey literature were searched. Two reviewers independently screened reports, extracted data, and performed risk of bias assessment. Implicit interventions of interest were analogy-, errorless-, dual-task-, and external focus learning. Data analysis involved descriptive synthesis of group comparisons on absolute motor dual-task (DT) performance, and motor DT performance relative to single-task motor performance (motor DTCs).

**Results:**

Of the 4125 reports identified, we included 25 controlled trials that described 39 implicit-explicit group comparisons. Risk of bias was unclear across trials. Most comparisons did not show group differences. Some comparisons showed superior absolute motor DT performance (N = 2), superior motor DTCs (N = 4), or both (N = 3) for the implicit compared to the explicit group. The explicit group showed superior absolute motor DT performance in two comparisons.

**Conclusions:**

Most comparisons did not show group differences in automaticity. The remaining comparisons leaned more toward a greater degree of movement automaticity after implicit learning than explicit learning. However, due to an overall unclear risk of bias the strength of the evidence is level 3. Motor learning-specific guidelines for design and especially reporting are warranted to further strengthen the evidence and facilitate low-risk-of-bias trials.

## 1. Introduction

The prospect for enhancing motor skill learning is exhilarating for practitioners in sports, rehabilitation, and physical education. Accordingly, when implicit learning interventions were proposed in handbooks of coaching and sport psychology[1–4] as alternative to traditional explicit instruction-based learning methods, these were readily adopted in sports practice (e.g. football,[5] soccer,[6] and baseball[7]). The more traditional methods presume that motor learning necessarily progresses from an initial verbal-cognitive phase, during which a learner gains declarative knowledge about the technicalities of movement skill (i.e., regularities and facts of movement execution) to increase performance, to a final autonomous phase, in which the skill has become an automatized, procedural routine and the learner is barely aware of movement execution.[8,9] This mode of learning is generally referred to as *explicit* learning: “… learning which generates verbal knowledge of movement performance (e.g. facts and rules), involves cognitive stages within the learning process and is dependent on working memory involvement” [10] (18, p.5).

**Fig 1:**
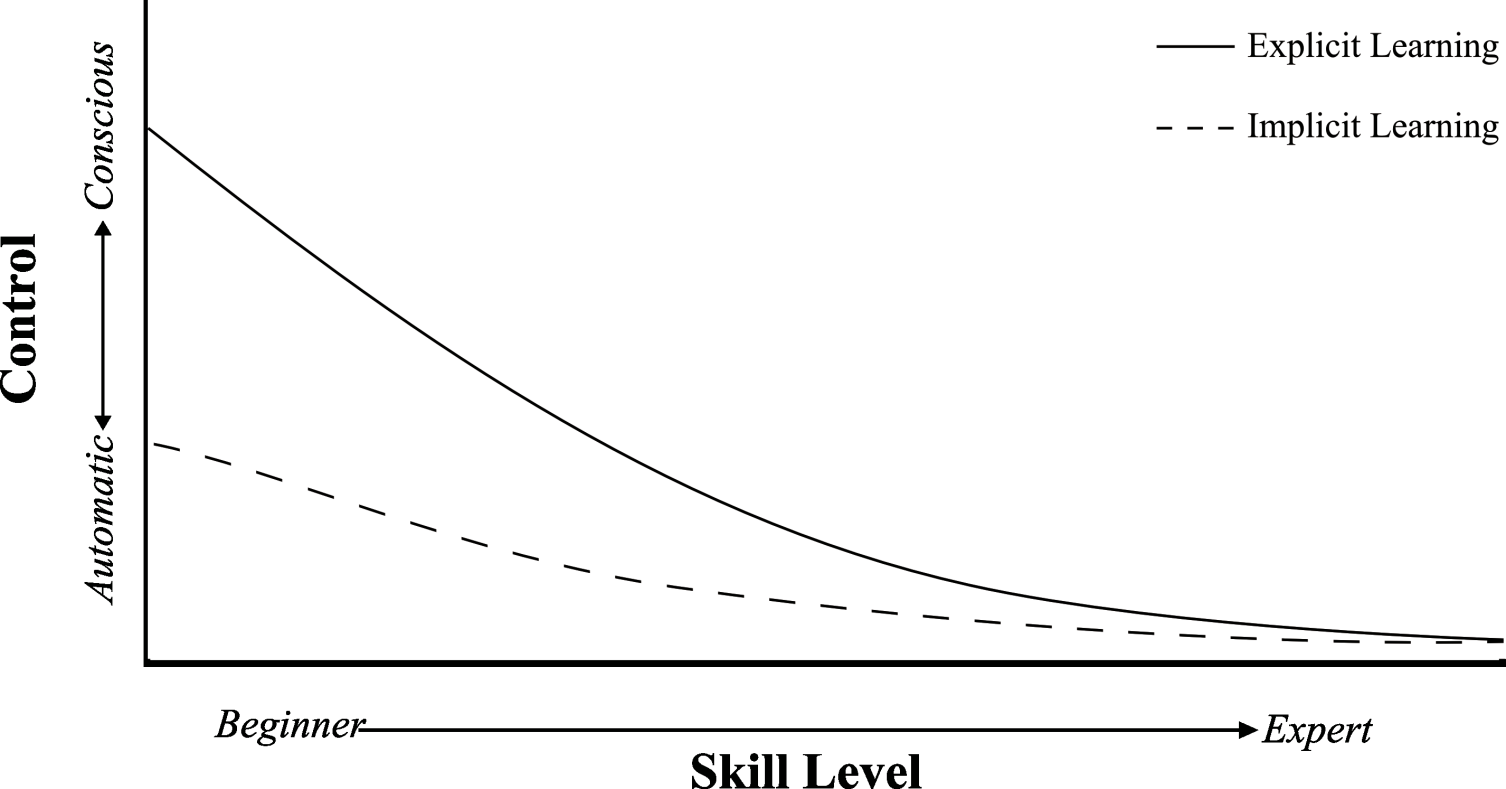
Schematic representation or relation between implicit and explicit motor learning and conscious control/automatic control as a function of skill level. With explicit learning (solid line), motor control is highly cognitively demanding at the start of learning (in what Fitts and Posner called the verbal-cognitive stage). With implicit learning (dashed line), motor control is relatively less dependent on conscious control, and hence more automatic right from the start of learning. As skill acquisition unfolds both explicit and implicit learning will result in more and more automated motor control, and eventually converge. By measuring dual-task performance the degree of automaticity achieved can be measured.[12,14,15] Please note that the model also takes into account that skill level and automaticity are tightly related, but not interchangeable entities (i.e., skill acquisition involves more than just automating motor control).[16,17] Thus, for the same level of skill, performers may substantially differ in terms of the degree of conscious/automatic control involved. That said, skill level and automaticity generally co-develop with practice. Hence, skill level is an important confounder when assessing automaticity of movement.

By contrast, implicit learning methods take as starting point that such an initial cognitive phase of declarative knowledge accrual is not mandatory. Instead, motor skill acquisition would involve direct accumulation of procedural knowledge, which is inaccessible for consciousness and is not dependent on working memory processing. Learners generally are unable to verbally describe the technicalities of the skill.[1,10–12] Thus, motor skills that are learned implicitly are thought to be less reliant on declarative knowledge compared to skills that are learned explicitly,[11] and instead more strongly capitalize on automatic processes. [10,13] In other words, after implicit learning motor control should be characterized by a greater degree of automaticity or, since they are two sides of the same coin, by reduced conscious control. This should be particularly evident in early learning, given that with protracted practice also explicit motor learning should eventually culminate in automatized motor control (see Fig 1).

Automatized motor skills are less easily disturbed when the performer’s cognitive resources are compromised, for instance due to fatigue, psychological pressure, or when concurrent tasks are performed. Assessment of dual-task performance has typically been exploited by researchers to examine the degree of movement automaticity achieved, or conversely, the degree to which conscious control is still required for performance.[11–13,15,18] The tenet is that the degree of automaticity is proportional to the disruption caused by cognitively demanding dual-tasks: The more automatized the motor skill, the more robust performance is in dual-task conditions.[14] A critical prediction therefore is that implicit learning results in superior dual-task performance compared to explicit motor learning, already after short practice periods.

The presumed greater automatization of motor skills after implicit motor learning bears great significance. In sports, maintaining performance in the face of highly demanding dual-task situations is key to success (e.g., simultaneously monitoring game tactics and hitting a drop shot in tennis) and might even diminish risk of (re-)injury.[19,20] Moreover, motor performance should be more resilient to break down in fatiguing or high pressure situations[1]–i.e., when the athlete does not accumulate explicit knowledge early in learning, he/she will be less likely to de-automatize motor performance by falling back on (or “reinvest”) such knowledge in these situations.[21] Hence, implicit motor learning methods have gained increasing interest among sport coaches. It is recommended in handbooks of sport psychology[3,4,22] and implicit motor learning principles are now increasingly applied in (inter-)national sports (e.g. football,[5] soccer,[6] and baseball[7]). Similar developments have been signaled in rehabilitation.[23–25]

Given its potential significance to sports science and practice, it is important to verify whether implicit motor learning indeed results in a greater degree of movement automaticity relative to explicit learning. Although individual research papers seem to support this claim, a systematic review is lacking. Hence, our aim here was to perform a comprehensive systematic review comparing the degree of movement automatization achieved after implicit and explicit motor learning interventions of sports tasks in healthy adults. Automaticity of movement was operationalized as motor skill performance during dual-tasking, probed on a separate test after the explicit or implicit learning interventions were terminated. Two aspects of dual-task performance were investigated, namely (1) absolute motor performance in dual-task conditions and (2) the robustness of motor performance to dual-task interference (i.e., the relative difference in performance between single- and dual-task conditions, so-called motor dual-task costs). If implicitly learned skills are indeed more automatic we should find higher absolute motor dual-task performance and lower motor dual-task costs for the implicit groups compared to explicit groups. In addition to summarizing the evidence, we performed a risk of bias assessment to assess the certainty that there were no systematic factors that distorted the implicit-explicit comparisons in the included studies. This is imperative for reliable evaluation of results, as higher risk of bias leads to less reliable effect estimates, especially in light of recent reports of issues with bias in motor learning research in general.[26,27]

## 2. Methods

Prior to our search we registered our review on PROSPERO (International prospective register of systematic reviews; registration number CRD42016038249).

### 2.1. Criteria for inclusion of studies

#### 2.1.1. Population

Studies that investigated healthy athletes/adults (>18 years of age) were included. Studies that included athletes with non-neurological sports-related injuries (e.g., ankle sprain, knee injury) were also eligible for selection.

#### 2.1.2. Experimental design

Studies were included if they compared the effects of an implicit- with an explicit motor learning intervention on motor task performance in single- and dual-task (motor-motor or motor-cognitive) conditions on separate retention tests (i.e., after practice was terminated and the experimental interventions were no longer provided). Such tests are imperative to determine whether an intervention has any lasting effect on motor performance and automaticity. In motor learning literature, retention tests often refer to single-task motor performance assessments while dual-task assessments are generally referred to as “transfer tests”. In this review we use the terms “retention tests/at retention” to indicate the point in time that assessments took place (i.e., after practice was terminated), rather than the type of assessment (single-task vs. dual-task).

We distinguished between studies with immediate (<24h) and delayed (>24h) retention tests.[28,29] Published and non-published controlled trials for which a full report was available were eligible for inclusion.

#### 2.1.3. Implicit and explicit motor learning interventions

Studies were included if they compared explicit and implicit motor learning interventions. This review followed the definitions outlined by a recent Delphi study,[10] which we also used in an earlier review on implicit motor learning post-stroke.[26] As such, implicit and explicit motor learning are thus not necessarily considered to be separate processes, but rather as two ends of a continuum, with purely implicit motor learning on one end (motor performance occurs without any processing of declarative movement related knowledge in working memory) and purely explicit motor learning on the other end (motor performance is completely dependent on the processing of declarative movement related knowledge in working memory). In sports practice, it will be difficult to induce pure implicit motor learning, as athletes will always have some awareness and verbal knowledge of their performance. Yet, interventions may lead to relatively more implicit learning when they actively minimize athletes’ use of explicit declarative knowledge to improve their performance.

Hence, the following motor learning interventions were labeled as ‘implicit’: (1) Analogy learning: Providing the learner with a metaphorical instruction that summarizes the global structure of the skill, and consequently only requires minimal conscious processing on the part of the learner (e.g., for basketball free throws: “Shoot as if you are trying to put cookies into a cookie jar on a high shelf”[30]); (2) Errorless or error-reduced learning: Minimizing errors during practice (e.g., initially practice golf putting at close range, and then gradually increase putting distance[31]). The idea is that when no (or few) errors occur, the learner will not be enticed to test hypotheses how to improve performance–which is a highly cognitively demanding strategy; (3) Dual-task learning: Performing a concurrent attention-demanding secondary task during practice. A concurrent secondary task utilizes working memory capacity, thereby restricting a learner’s opportunity to consciously control his or her movements (e.g., randomly generating letters while performing a table tennis forehand[32]); (4) External focus learning[33]: The idea is that when learners focus attention on movement effects/goals, this reduces interference of normal automatic (implicit) motor control compared to when they focus attention internally on the body and biomechanics of movement (e.g., for dart throwing: focusing on the flight of the dart or the bull’s eye, rather than on the movements of the hand [34]).

In contrast, verbal explicit instructions (that describe how the participant should perform the movement), errorful learning/trial-and-error learning, and internal focus learning (where the learner is instructed to focus on movement execution itself) were considered to be ‘explicit’ motor learning strategies.[10] So-called “discovery learning” interventions were only included as an explicit intervention if learners were explicitly instructed to actively search rules of movement.

#### 2.1.4. Types of motor tasks

Classical studies into implicit motor learning have focused on the sequencing processes underlying motor learning, by having participants learn a sequence of button presses (i.e., the serial-reaction time” (SRT) paradigm). Learning sports-related tasks, however, typically requires one to acquire and optimize the dynamics of movement rather than to master the appropriate sequence of movement.[25] Therefore, we only included studies in which participants needed to learn tasks with relatively complex movement dynamics (e.g., throwing, kicking, jumping, grasping, balancing, and the like), while excluding studies that merely focused on sequence (SRT) learning.[25] Also, since performing as good as possible is a key element in sports, we considered motor tasks to be sports-related only when such a performance optimizing criterion was given.

#### 2.1.5. Outcome measures

In order to make a consistent comparison between learning interventions, we only focused on (dual-task) *performance* measures (e.g. seconds, meters, percentages, etc.). The degree of automaticity of movement was operationalized as motor dual-task performance after practice was completed. Two aspects of dual-task performance were assessed: (1) absolute motor performance in dual-task conditions and (2) the robustness of motor performance to dual-task interference (i.e., the relative difference in performance between single- and dual-task conditions, so-called motor dual-task costs).[35]

### 2.2. Data sources and searches

A medical research librarian assisted in the formulation of our search strategy (see S1 Text). We did not impose any restrictions to our search. Two investigators (RP and EK) searched the following electronic databases, from their inception up till March 2^nd^ 2017: MEDLINE (via Pubmed), CENTRAL, Embase, PsycINFO, SportDiscus and Web of Science. Unpublished reports, conference abstracts, ongoing studies and other grey literature were searched in BIOSIS Previews, British Library Inside, OpenGrey.eu, Clinical Trials.gov, The European Union Clinical Trials Register, ISRCTN registry, and the WHO International Clinical Trials Registry Platform.

### 2.3. Study selection

First, study eligibility was assessed based on title and abstract. Potential relevant reports were further assessed based on full text. The selection process was performed by two reviewers independently (RP and EK). In case of disagreement, reviewers sought consensus through discussion. A third independent reviewer (JK) was consulted in case of persistent disagreement.

### 2.4. Data extraction

Two reviewers (RP and EK) independently extracted data by means of a standardized data extraction form. We extracted information regarding design, methodology, demographics (e.g., age, gender, skill level, cognitive function tests); information regarding the experimental- and control intervention (e.g., type of motor learning intervention; frequency, volume, and duration of practice, retention test interval, type of dual-task); outcome measures and findings (estimates and measures of dispersion).

### 2.5. Risk of bias assessment

As stated earlier, for systematic reviews to obtain reliable conclusions, it is pertinent that potential limitations of the included studies are considered carefully. We used the Cochrane’s risk of bias tool for this purpose.[36] Two reviewers (one with expertise in motor learning, EK, and one epidemiologist, MW) independently evaluated the 5 major domains of biases (see Cochrane for detailed information[36]):

- Selection bias: i.e., the presence of systematic differences between experimental groups in terms of possible confounding prognostic factors. This can be prevented by proper random allocation of participants to an experimental and control group, and by concealing the allocation from the persons involved in participant enrollment.
- Performance bias: i.e., the presence of systematic differences between groups in how interventions are administered, other than the differences between the experimental and control intervention. Think of more, longer, or more intense practice sessions for one group compared to the other, or of differences in exposure to other important factors (e.g., the person providing the intervention may implicitly have a more positive attitude towards and/or gives more attention to one group of participants than to the other. This can be prevented by blinding the participants and personnel providing the intervention to group allocation)
- Detection bias: i.e., a systematic difference in how the intervention’s outcome is determined. This may especially influence subjective outcomes (e.g., the outcome assessors beliefs/hypotheses regarding the interventions of interest may implicitly make him/her more likely to award higher points to one group than to the other), but also plays a role with objective outcomes (e.g., when the assessor (implicitly) has a more positive attitude toward one group of participants than to the other, this may systematically influence performance outcomes). Detection bias can be prevented by blinding the outcome assessor
- Attrition bias: i.e., the presence of systematic group differences in the number of persons that quit or drop-out from the experiment prematurely, or that are excluded from analyses. Attrition bias is low when a study accurately reports study flow, and there are no clear imbalances between groups in terms of drop-outs or exclusions.
- Reporting bias: i.e., the presence of differences between the reported (published) findings, and the initially planned and/or non-reported analyses. Low risk of reporting bias can be ascertained when a registered study protocol confirms that all analyses were carried out as planned, and all planned outcomes have been reported.
- Other bias: We additionally determined whether there were any other potential risk of biases, such as the absence of a separate pretest to assess possible baseline differences in motor ability between groups

Two reviewers (one with expertise in motor learning, EK, and one clinical epidemiologist, MW) independently evaluated the included studies. Risk of bias on each domain was scored using a set of predefined criteria. In line with recommendations, we specifically modified these criteria for the purpose of this review (S1 Table).[36,37] Individual items were scored '+' for low risk of bias; '-' for high risk of bias and '?' for unclear risk of bias. Eventually, controlled trials were classified as low risk of bias (all items: ‘+’), moderate risk of bias (1 or 2 items: ‘-’), or high risk of bias (>2 items: ‘-‘). Trials were assigned an unclear risk of bias when 4 or more items were scored ‘?’.

We scored the corresponding overall 'Level of Evidence' in accordance to the table of Oxford's Centre for Evidence-Based Medicine.[38] In this system, level 1 evidence is assigned to systematic reviews. Randomized controlled trials at low risk of bias are classified as level 2 of evidence. Lastly, nonrandomized controlled trials are assigned a level 3 evidence. In case of an overall unclear or high risk of bias the strength of the evidence may be reduced by 1 level.[38]

### 2.6. Data synthesis and analysis

The analysis focused on both absolute dual-task performance and dual-task costs at retention. For absolute performance, we assessed performance of the primary, newly-learned motor skill and the secondary task during dual-task conditions. To determine dual-task costs, we calculated the percentage difference in performance between single-task (ST) and dual-task (DT) conditions at retention using the following formula: DT costs (DTC) = [(ST-DT)/ST*100]. Higher costs indicate a larger deterioration of performance in the DT condition compared to the ST condition. If possible, we also calculated DT costs for the secondary task. Secondly, we assessed the reported amount of declarative knowledge of the intervention- and control groups, to assess the degree to which the interventions induced explicit (and thus implicit) learning. If the implicit group had gained significantly less declarative knowledge than the explicit group, the manipulation was considered successful.[1,10,11,26]

We planned a meta-analysis when studies were sufficiently homogeneous and study outcomes were at low risk of bias. In the presence of an overall unclear or high risk of bias it’s recommended to refrain from pooling data in a meta-analysis. [36] Accordingly, in the case that data synthesis would turn out to be inappropriate we planned a descriptive synthesis. When data of interest was not specifically reported in the text, extraction of outcome values would be done, if necessary, manually (i.e., conversion from graphs using InkScape). Subsequently, we conducted an unpaired independent t-test if the exact relevant means and standard deviations could be obtained. If not, P-values for the comparisons of interest were extracted from the studies' text. Finally, a funnel plot was used to assess the possible presence of publication bias.

## 3. Results

### 3.1. Study selection

**Fig 2:**
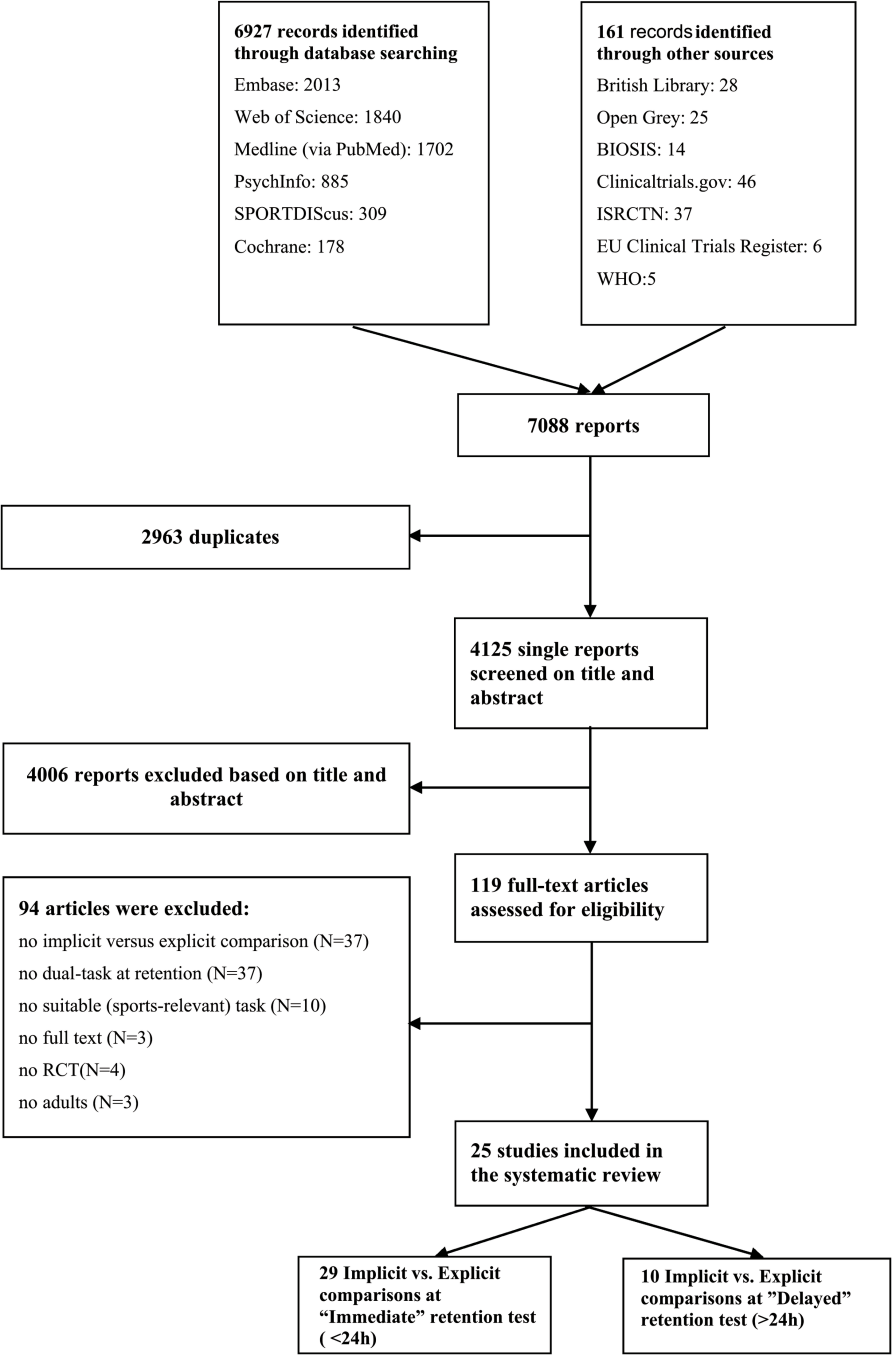
Flow chart of study search and selection.

Fig 2 shows the flow of study selection. The search yielded a total of 4125 single hits. Screening for title and abstract resulted in the identification of 119 possibly relevant reports. However, a majority of these reports was excluded after full text screening because they did not make a comparison between implicit- and explicit learning interventions (N = 37), or lacked dual-task assessment at retention (N = 37). Nine other studies were excluded because they did not investigate a sports-relevant (motor) task. Three congress abstracts were identified that were possibly relevant. However, attempts to contact the primary investigators were unsuccessful.

Eventually, 25 studies were included in this systematic review (Fig 2).[13,15,30–32,39–58] Several studies described multiple experiments (N = 4[31,46,50,53]), retention tests (i.e., both immediate and delayed retention tests; N = 3[41,47,53]), or intervention groups (i.e. two implicit groups; N = 6[13,31,32,40,47,58]). We evaluated these separately, such that our review includes a total of 39 implicit-explicit motor learning comparisons: 29 concern comparisons on immediate retention tests, and 10 concern comparisons on delayed retention tests. The possibility of publication bias was explored by means of a funnel plot (S1 Fig). Only half (N = 19) the comparisons could be included in the funnel plot, as standard deviations were missing for the other studies. No evidence for publication bias was deemed present: The funnel plot appeared to have a symmetrical distribution, and Egger’s[59] test revealed that the distribution was not statistically asymmetrical (*B*_0_ = -1.821,SE = 1.192, 95% CI[-4.335, 0.693], p = 0.145).

### 3.2. Study characteristics

S2 Table provides a detailed overview of each study’s characteristics. Twenty-five randomized controlled trials were included, totaling 1040 participants (41% men vs. 59% women). Most studies concerned young adults (mean age = 26.50 years; range = 18–67 years), who were novice with respect to the motor task they needed to learn: 20 of the 25 studies explicitly state that participants had no prior experience, 5 studies do not describe participants’ experience. Overall the majority of studies involved small sample sizes (mean = 13.9 participants per experimental group, range = 6–25). Practice durations varied from 1 day to 6 weeks, while subsequent retention interval ranged from 5 minutes to 2 weeks. The types of motor tasks investigated included: golf tasks (N = 6[13,31,49,50,54,55]), table tennis tasks (N = 6[32,40,41,43,52,58]), balance board tasks (N = 4[15,46–48]), basketball free throws (N = 2[30,42]), rugby passing (N = 2[45,51]), miscellaneous aiming/throwing tasks (N = 3[39,53,56]), and a surgical task[44] and Pedalo riding[57] (both N = 1). Dual-task assessments at retention mostly consisted of counting tasks; both backward counting (N = 8[30,32,39–41,52,57,58]) and tone counting (N = 6[13,31,46,49,50,53]) were frequently tested. Other studies used (variants of) probe reaction tasks (N = 5[15,42,43,54,55]) or had participants generate random digits/letters or sequences thereof (N = 6[44,45,47,48,51,56]). Only one study explicitly mentioned that participants had to prioritize motor task performance over secondary cognitive task performance.[15]

### 3.3. Risk of bias assessment

The risk of bias assessment was performed separately for each experiment. In this section, we therefore refer to experiments, rather than studies.

**Fig 3:**
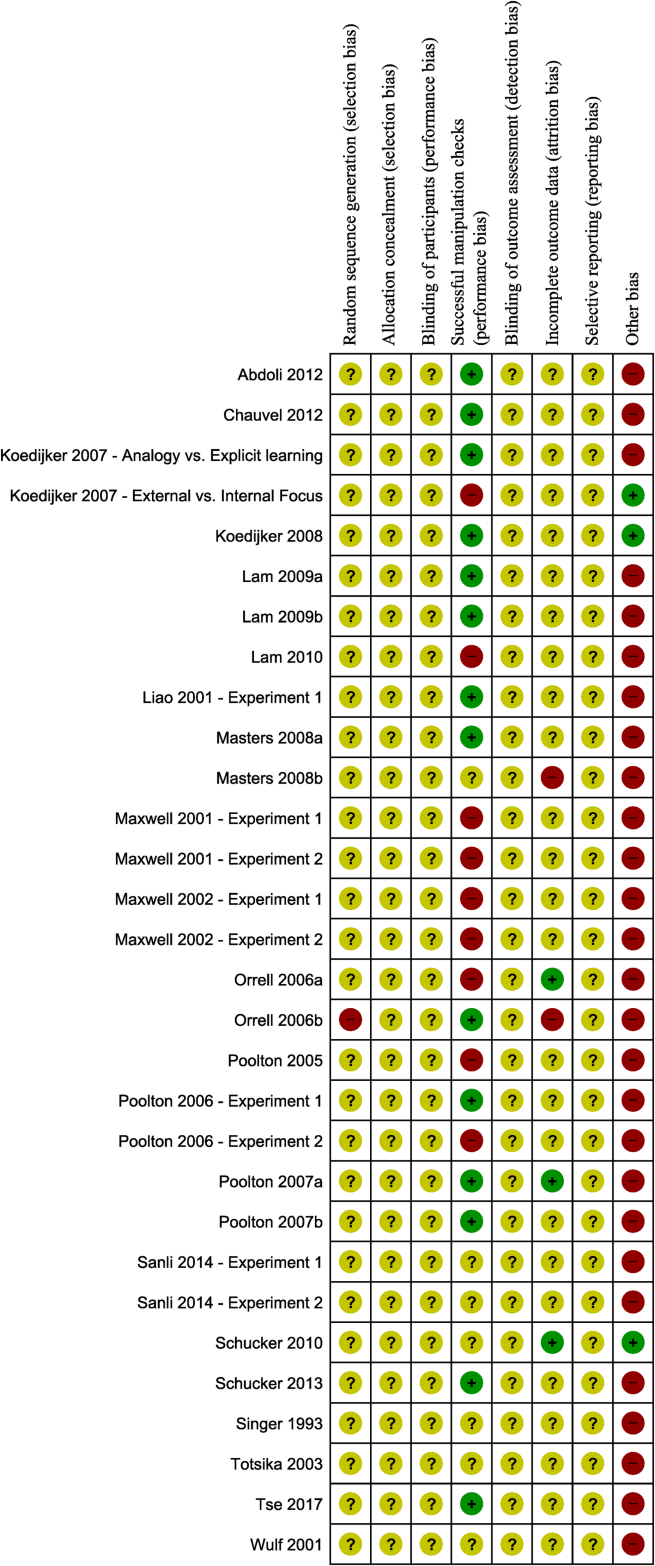
Summary of risk of bias assessment per experiment. NB: ‘-’ is high risk of bias; ‘+’ = low risk of bias; ‘?’ = unclear risk of bias.

Fig 3 provides an overview of biases per domain per experiment. Overall, experiments exhibited an unclear risk of bias. This was predominantly due to a significant lack of reporting. For instance, no detailed descriptions were available of randomization procedures (but see [47]) and blinding of researchers, participants and outcome assessor, nor were any study protocols available to assess reporting bias. Further, only 5 experiments reported on the number of drop-outs in the experiment.[45,47,48,51,54] Of these, 2 experiments[45,47] scored a high risk of so-called attrition bias, due to a drop-out rate of more than 10%.

Generally, experiments scored best on one item of performance bias, namely the check the degree to which learning was indeed more implicit in the implicit learning group than in the explicit learning group. In 14 experiments the implicit group reported less explicit knowledge than the explicit group,[13,30,32,39–42,44,47,50–52,55,58] while this was not the case for 9 other experiments.[31,40,43,46,48–50] Seven experiments lacked this manipulation check, and therefore were scored as having an “unclear risk of bias” on this item.[15,45,53,54,56,57]

In the category other biases it was assessed whether groups were of similar motor skill before the intervention. For 3 experiments no differences in motor skill were evident on a pretest, and these were thus scored as having a low risk of bias on this domain.[40,41,54] All other studies were assigned a high risk of bias. With regard to the use of a pretest, we do acknowledge that there is a good reason for researchers to not incorporate a pretest in their design. That is, during a (task-specific) pretest learners may already acquire explicit knowledge of the to-be-learned motor skill, which would interfere with subsequent implicit motor learning.[31] However, please note that the overall risk of bias assessment would be unaffected and remain “unclear”, even if the absence of pretest assessments would not be taken into consideration. We refer to the discussion section for a more detailed discussion of this and the other risk of bias issues noted here.

Overall, all studies were generally found to be at unclear risk of bias. This meant that (1) the strength of the evidence was confined to level 3 (nonrandomized controlled trials); and (2) that descriptive data synthesis was performed, as data synthesis by means of meta-analysis was not justified.[36]

### 3.4. Descriptive synthesis

Tables 1 and 2 give an overview of the main intervention effects for all experiments and comparisons made. Based on retention interval, we made a distinction between immediate (<24h; Table 1) and delayed (>24h; Table 2) retention test phases. We categorized different comparisons of learning interventions to discuss our main outcome values: absolute (motor + cognitive) dual-task (DT) performance; (motor + cognitive) dual-task costs (DTC); declarative knowledge. In order to show strong evidence for superior DT performance due to implicit motor learning, we determined that the implicit group must demonstrate significantly better motor DT performance (i.e., better absolute motor DT performance or lower motor DTCs) *and* significantly less declarative knowledge compared to the explicit group.

**Table 1:** Summary of intervention effects for comparisons with immediate (<24h) retention intervals.

Study/experiment	Comparison	Significant group differences (implicit vs explicit group)					
		*Motor ST*	*Motor DT*	*Cognitive DT*	*Motor DTC*	*Cognitive DTC*	*Declarative knowledge*
Chauvel et al. (2012) [13]	Errorless vs Errorful—Young	=	=	=	N/A	N/A	+
	Errorless vs Errorful—Old	=	=	=	N/A	N/A	+
Lam et al. (2010) [43]	Errorless vs Errorful	=	=	=	=	N/A	=
Masters et al. (2008b)[45]	Errorless vs Errorful	=	+	N/A	+	N/A	N/A
Maxwell et al. (2001)—*Experiment 1* [31]	Errorless vs Errorful	=	+	=	+	N/A	=
Maxwell et al. (2001)—*Experiment 2* [31]	Errorless vs Errorful	?	?	=	N/A	N/A	=
Poolton et al. (2005) [49]	Errorless vs Errorful	?	?	=	+	N/A	=
Poolton et al. (2007a) [51]	Errorless vs Errorful	=	=	N/A	+	N/A	+
Sanli et al. (2014)—*Experiment 1-Test Phase 1* [53]	Errorless vs Errorful	=	=	=	=	N/A	N/A
Sanli et al. (2014)—*Experiment 2-Test Phase 1* [53]	Errorless vs Errorful	=	=	=	=	N/A	N/A
Masters et al. (2008a) [44]	Errorless vs Explicit	=	=	N/A	=	N/A	+
Orrell et al. (2006a) [47]	Errorless vs Explicit	=	=	N/A	=	N/A	=
Orrell et al. (2006b)—*Test phase 1* [48]	Errorless vs Explicit	=	=	N/A	=	N/A	+
Koedijker et al. (2007) [40]	Analogy vs Explicit	=	=	=	=	N/A	+
Koedijker et al. (2008)—*Test phase 1* [41]	Analogy vs Explicit	=	=	=	=	N/A	+
Liao et al. (2001)—*Experiment 1* [32]	Analogy vs Explicit	?	?	N/A	+	N/A	+
Orrell et al. (2006b)—*Test phase 1* [48]	Analogy vs Explicit	-	-	N/A	=	N/A	+
Poolton et al. (2007b) [52]	Analogy vs Explicit	?	?	N/A	+	N/A	+
Schücker et al. (2010) [54]	Analogy vs Explicit	=	=	=	?	N/A	N/A
Schücker et al. (2013) [55]	Analogy vs Explicit	?	=	=	N/A	N/A	+
Tse et al (2017) [58]	Analogy vs Explicit–Young	+	+	N/A	=	N/A	+
	Analogy vs Explicit–Old	+	+	N/A	=	N/A	+
Koedijker et al. (2007) [40]	External vs Internal	=	=	=	=	N/A	=
Maxwell et al. (2002)—*Experiment 1*[46]	External vs Internal	=	=	=	=	N/A	=
Maxwell et al. (2002)—*Experiment 2* [46]	External vs Internal	=	=	=	=	N/A	=
Poolton et al. (2006)—*Experiment 1* [50]	External vs Internal	=	+	=	+	N/A	+
Poolton et al. (2006)—*Experiment 2* [50]	External vs Internal	=	=	=	=	N/A	=
Singer et al (1993) [56]	External vs Internal	?	+	=	N/A	N/A	N/A
Liao et al. (2001)—*Experiment 1* [32]	Dual-task vs Explicit	?	?	N/A	+	N/A	+

**NB:** Green ‘+’: Significantly (p<0.05) better performance or less declarative knowledge for implicit group compared to explicit group; Yellow ‘-’: Significantly (p<0.05) better performance or more declarative knowledge for explicit group compared to implicit group; ‘ = ‘: No significant difference between implicit and explicit groups; ‘?’: Outcome measure was assessed, but corresponding p-values could not be obtained; N/A: Outcome measure not assessed. Abbreviations: DT = dual-task; DTC = dual-task costs.

**Table 2:** Summary of intervention effects for comparisons with delayed (>24h) retention intervals.

Study/Experiment	Comparison	Significant group differences (implicit vs explicit group)					
		*Motor ST*	*Motor DT*	*Cognitive DT*	*Motor DTC*	*Cognitive DTC*	*Declarative knowledge*
Abdoli et al. (2012) [39]	Errorless vs Errorful	+	+	=	+	N/A	+
Sanli et al. (2014)—*Experiment 1-Test Phase 2* [53]	Errorless vs Errorful	=	=	=	=	N/A	N/A
Sanli et al. (2014)—*Experiment 2-Test Phase 2* [53]	Errorless vs Errorful	=	=	=	=	N/A	N/A
Orrell et al. (2006b)—*Test phase 2* [48]	Errorless vs Explicit	=	=	=	?	N/A	+
Koedijker et al. (2008)—*Test phase 2* [41]	Analogy vs Explicit	=	=	=	=	N/A	+
Lam et al.(2009a) [42]	Analogy vs Explicit	=	=	=	=	N/A	+
Lam et al.(2009b) [30]	Analogy vs Explicit	=	+	=	+	N/A	+
Orrell et al. (2006b)—*Test phase 2* [48]	Analogy vs Explicit	-	-	=	?	N/A	+
Totsika et al. (2003) [57]	External vs Internal	+	+	N/A	?	N/A	N/A
Wulf et al. (2001) [15]	External vs Internal	+	+	+	=	+	N/A

**NB:** Green ‘+’: Significantly (p<0.05) better performance or less declarative knowledge for implicit group compared to explicit group; Yellow ‘-’: Significantly (p<0.05) better performance or more declarative knowledge for explicit group compared to implicit group; ‘ = ‘: No significant difference between implicit and explicit groups; ‘?’: Outcome measure was assessed, but corresponding p-values could not be obtained; N/A: Outcome measure not assessed. Abbreviations: DT = dual-task; DTC = dual-task costs.

Finally, where possible, we also report single-task (ST) results for each group comparison. This was done to check whether differences in motor skill level possibly confounded group comparisons in dual-task performance. For instance, less skilled ST motor performance may result in greater decrements in motor performance in DT conditions [60] (see also Fig 1).

We refer to the ‘Results’ section of S2 Table for details on the extracted data for each comparison.

#### 3.4.1. Immediate retention (<24h)

First, we describe the results of the thirteen comparisons of errorless and errorfull/explicit motor learning interventions. These comparisons concerned the following motor tasks: golf-putting (N = 5),[13,31,49] rugby-throwing (N = 2),[45,51] disc-propelling (N = 2),[53] a surgical task (N = 1),[44] balancing (N = 2),[47,48] and table tennis.[43] The DT assessments consisted of tone-counting (N = 7),[13,31,49,53] probe reaction time(N = 1),[43] random letter generation (N = 3),[47,51] and digit sequence recall plus kettle lift (N = 2).[48]

No single comparison showed significant differences in motor ST performance for the implicit (i.e., errorless) compared to the explicit group.

Two comparisons[31,45] found significantly better motor DT performance for the implicit group compared to the explicit group. Nine comparisons[13,43,44,47,48,51,53] did not show significant differences, whereas this measure could not be obtained for two other comparisons.[31,49] Cognitive DT performance did not differ for eight comparisons[13,31,43,49,53] whereas this measure was unavailable for the other five comparisons.[44,45,47,48,51] Four comparisons[31,45,49,51] revealed significantly lower motor DTC for the implicit group. Six comparisons[43,44,47,48,53] did not show significant differences, whereas no motor DTCs were available for other three comparisons.[13,31] No comparisons were available for cognitive DTCs.

Five comparisons[13,44,47,51] found significantly less declarative knowledge for the implicit group, five others[31,43,48,49] did not reveal any group differences, while the three others[45,53] did not assess this measure.

Combined, no comparison showed superior absolute motor DT performance paired with less declarative knowledge for the errorless group compared to the explicit group, while one comparison showed superior motor DTCs and less declarative knowledge for the errorless group compared to the explicit group.[51] Thus, there is little evidence that errorless learning benefits motor DT performance compared to explicit learning.

Second, we included nine comparisons of analogy versus explicit motor learning. These concerned the following motor tasks: table-tennis (N = 6),[32,40,41,52,58] balancing (N = 1),[47] and golf (N = 2).[54,55] DT assessments included counting backwards (N = 6),[32,40,41,52,58] digit sequence recall plus additional kettle lift (N = 1),[47] or a tone-judgment task (N = 2).[54,55]

With regard to single-task motor performance, group differences were evident for three comparisons. Tse et al. found significantly better ST performance for the implicit (i.e., analogy) groups (both young and old) than for the explicit groups,[58] while Orrell et al. reported opposite results.[47]

Tse et al.[58] found significantly better motor DT performance for the implicit groups than for the explicit groups, both in young and older adults. In contrast, Orrell et al.[47] found significantly better motor DT performance for the explicit- in comparison to the implicit group. Four comparisons[40,41,54,55] did not show significant differences, whereas this measure could not be assessed for two other comparisons.[32,52] In four comparisons[40,41,54,55] cognitive DT performance did not significantly differ, whereas for the other five[32,47,52,58] this measure was not assessed. Two comparisons[32,52] showed significantly lower motor DTC for the implicit group. Five comparisons[40,41,47,58] did not show significant differences, whereas this measure was not available for two other comparisons.[54,55] None of the experiments analyzed DTC for the cognitive task.

For eight comparisons[32,40,41,47,52,55,58] significantly less declarative knowledge was reported by the implicit group than by the explicit group. Schücker et al.[54] did not asses this measure.

Combined, two comparisons reported superior absolute motor DT performance combined with less declarative knowledge for the analogy group compared to the explicit group,[58] while one comparison found *inferior* absolute motor DTs and less declarative knowledge for the analogy group.[47] Two comparisons revealed superior motor DTCs and less declarative knowledge for the analogy compared to the explicit groups.[32,52] Thus, there is weak evidence that analogy learning benefits motor DT performance compared to explicit learning.

Third, we included six external focus vs. internal focus comparisons. These concerned the following motor tasks: table-tennis (N = 1),[40] balancing (N = 2),[46] golf (N = 2),[50] and ball throwing (N = 1).[56] DT assessments ranged from counting backwards (N = 1)[40] and tone-counting (N = 4)[46,50] to digit sequence recall (N = 1).[56]

No single comparison showed significant differences in ST motor performance for the implicit (i.e., external) compared to the explicit (i.e., internal) group.

Two comparisons[50,56] revealed significantly better motor DT performance for the implicit- in comparison to the explicit group. The remaining four comparisons did not show any group differences in motor DT performance.[40,46,50] Cognitive DT performance was similar across groups for all six comparisons. Poolton et al. (Experiment 1)[50] reported significantly lower motor DTC for the implicit group. Four comparisons[40,46,50] did not show significant differences, whereas Singer et al.[56] did not assess this measure. No comparisons were available for cognitive DTCs.

Poolton et al. (Experiment 1)[50] found significantly less declarative knowledge for the implicit group. For four comparisons, no significant differences were found between groups.[40,46] Singer et al.[56] did not assess this measure.

Combined, one comparison[50] found that the external focus group showed superior absolute motor DT performance, superior motor DTCs, and reported less declarative knowledge compared to the explicit group. Thus, there is little evidence that external focus learning benefits motor DT performance compared to explicit learning.

The fourth comparison of interest was that of dual-task vs explicit motor learning interventions. Liao et al.[32] compared the effectiveness of these interventions on learning a table tennis task. The dual-task assessment consisted of counting backwards.

No absolute motor and cognitive single and dual-task performance measures could be obtained from the report. Significantly lower motor DTCs for the implicit group were reported. Cognitive DTCs were not reported. Liao et al.[32] did report significantly less declarative knowledge for the implicit group.

Thus, this comparison found evidence for better motor DT performance with implicit learning: the dual-task group showed both superior motor DTCs as well as less declarative knowledge compared to the explicit group.

#### 3.4.2. Delayed retention (>24h)

First, we included four errorless vs errorful/explicit instruction intervention comparisons. These concerned the following motor tasks: disc-propelling (N = 2),[53] ball-throwing (N = 1),[39] and balancing (N = 1).[47] DT assessments consisted of tone-counting (N = 2),[53] counting backwards (N = 1),[39] and tone-counting while kettle-lifting (N = 1).[47]

Abdoli et al.[39] reported significantly better ST motor performance for the implicit (i.e., errorless) than for the explicit group. The other three comparisons did not show any differences in single-task performance.[47,53]

Abdoli et al.[39] reported significantly better motor DT performance for the implicit group. The three other comparisons[47,53] did not reveal any group differences in motor DT performance. Implicit and explicit groups showed similar cognitive DT performance in all four comparisons. Abdoli et al.[39] reported significantly lower motor DTC for the implicit group. Two comparisons[53] did not show significant group differences in motor DTC, while motor DTCs were unavailable for one comparison.[47] Cognitive DTCs were lacking for all four comparisons.

Two comparisons[39,47] found significantly less declarative knowledge for the implicit- than for the explicit group. No information was available for the other two comparisons, because Sanli et al.[53] did not assess learners’ declarative knowledge.

Combined, out of the 4 comparisons, only Abdoli et al.,[39] reported superior absolute motor DT performance, motor DTCs, and less declarative knowledge for the errorless compared to the explicit group. There thus seems to be some evidence for better motor DT performance with errorless learning.

Second, we included four comparisons of analogy and explicit motor learning. These concerned the following motor tasks: basketball-throwing (N = 2),[30,42] table-tennis (N = 1),[41] and balancing (N = 1).[47] DT assessment consisted of counting backwards (N = 2),[30,41] probe reaction time (N = 1),[42] and tone counting while kettle-lifting (N = 1).[47]

Orrell et al.[47] reported worse ST motor performance for the implicit (i.e., analogy) group than for the explicit group. The other three comparisons did not find differences in ST motor performance.[30,41,42]

Lam et al.[30] found significantly better motor DT performance for the implicit group. Orrell et al.,[47] on the other hand, demonstrated significant better motor DT performance for the explicit group. The two remaining comparisons[41,42] did not show significant group differences in motor DT performance. None of the comparisons revealed significant differences in cognitive DT performance. Lam et al.[30] reported significantly lower motor DTC for the implicit group. Two comparisons[41,42] did not reveal significant differences, whereas this measure was unavailable for one other comparison.[47] Cognitive DTCs were lacking for all four comparisons.

All comparisons revealed significantly less declarative knowledge for the implicit group.

Combined, one comparison[30] reported superior absolute motor DT performance, superior motor DTCs, and less declarative knowledge for the analogy group compared to the explicit group, while one comparison[47] found inferior absolute motor DT performance, similar motor DTCs, and less declarative knowledge for the analogy group. Therefore, there is conflicting evidence that analogy learning benefits motor DT performance compared to explicit learning.

Finally, we included two studies that compared external with internal focus learning interventions. One comparison involved ‘Pedalo’ riding (N = 1)[57] and one a balance board task (N = 1).[15] The DT assessments were counting backward (N = 1)[57] and a probe reaction time task (N = 1).[15]

Both comparisons revealed[15,57] significantly better ST motor performance for the implicit- (i.e., external) than for the explicit (i.e., internal) group.

For both comparisons[15,57] significantly better motor DT performance was evident for the implicit group. Moreover, Wulf et al.[15] also showed a significantly better cognitive DT performance for the implicit group. This measure could not be obtained from Totsika et al.[57]

Wulf et al.[15] reported no significant differences in motor DTC, but did find lower cognitive DTC for the implicit group. Totsika et al.[57] did not assess motor and cognitive DTCs.

Both Wulf and Totsika did not assess[15,57] learners’ declarative knowledge.

Combined, there are clear indications for superior motor DT performance for the external focus groups than for the explicit groups. However, because declarative knowledge was not assessed, it is unclear whether this superior DT performance could be attributed to implicit motor learning.

## 4. Discussion

This systematic review assessed whether greater automatization of movement (or conversely, reduced reliance on conscious control) is achieved after implicit motor learning compared to explicit motor learning. This should be evidenced by implicit learning interventions resulting in superior absolute motor DT performance and/or lower motor DTCs, and less declarative knowledge compared to explicit interventions.

### 4.1. Main findings

In total, we included 25 controlled trials that described 39 implicit-explicit motor learning comparisons. In the majority of comparisons there were no group differences in absolute motor DT performance or motor DTCs. In 5 comparisons did the implicit group show superior absolute motor DT performance and less declarative knowledge compared to the explicit group.[30,39,50,58] In 7 comparisons lower DTCs and less declarative knowledge were found for the implicit group than for the explicit group.[30,32,39,50–52] Only in three comparisons did the implicit group show both significantly superior absolute DT performance *and* superior motor DTCs compared to the explicit group.[30,39,50] Opposite results were virtually absent, except for two comparisons which showed inferior absolute motor DT performance for the implicit group compared the explicit group.[47] No comparisons revealed better motor DTCs for the explicit group.

Those comparisons that found beneficial effects of implicit learning on motor DT performance involved different types of interventions–errorless learning,[39,51] dual-task learning,[32] analogy learning,[30,32,52,58] and external focus learning.[50] Also, these comparisons involved both immediate[32,45,50,52,58] and delayed[30,39] retention intervals. Thus, there are no strong indications that the effects of implicit motor learning on dual-task performance are influenced by the type of implicit intervention used, nor by retention interval. Yet, when we look at those comparisons for which ST motor performance results were also available, a trend is observed that ST and DT motor performance were correlated. That is, three of the six comparisons that showed better motor DT performance for implicit groups, also reported better ST motor performance[39,58] (with the other three not showing any ST differences[30,50,51]). Also, both comparisons that showed better DT performance for the explicit group also found better ST performance after explicit learning.[47] This raises the possibility that group differences in motor DT performance could in part be attributable to group differences in skill level per se, rather than the type of motor learning intervention (cf. Fig 1).

In sum, the majority of comparisons did not show differences in dual-task performance measures between implicit and explicit motor learning interventions. For the remaining comparisons there was a tendency toward better DT performance with implicit motor learning compared to explicit motor learning. As all studies scored an overall unclear risk of bias, the strength of the evidence is level 3. Below, we will first discuss how minimizing the risk of bias and more detailed reporting can strengthen motor learning research. We close with the implications for research and sports practice.

### 4.2. Minimizing risk of bias and strengthening research practices

The Cochrane risk of bias tool indicated an unclear risk of bias across the included studies, mostly due to underreporting of results. It thus seems that the expectations about reporting (and design) of authors, researchers, reviewers and editors in the field did not accord to the criteria used in the Cochrane risk of bias tool. To start with, we want to make absolutely clear that this must not be interpreted as an attack on the integrity of authors of the included studies, nor as evidence that the included studies were of poor quality. Also, we suspect that these findings are not specific to the studies in this review; earlier reviews revealed similar issues regarding underreporting and risk of bias issues in motor learning research in general.[26,27] Yet, the fact that we cannot establish the extent to which biases were *actually* present, or whether they affected the outcomes of the studies is precisely the main problem: It is impossible to tell whether the results summarized in this review are an accurate estimate of the underlying “true” effect, or whether they over- or underestimate it.[27] We therefore agree with Lohse et al.[27] that if the field of motor learning is to remain relevant, research and especially reporting practices need to be strengthened. Hence, the remainder of this section aims to increase awareness of the importance of detailed reporting and minimizing the risk of bias, and develop initial proposals to yield stronger levels of evidence. The risk of bias assessment performed in this review provides clear leads for this. These will be discussed in turn.

#### 4.2.1. Reporting bias

The main issue noted in this review is a serious lack of reporting. Therefore, first and foremost future studies should use the CONSORT[61] and STROBE[62] statements to ensure that researchers comprehensively describe their methods and results. In addition, study protocols should be registered in advance to improve transparency and prevent possible reporting bias. We acknowledge that up till now limited options were available to pre-register non-medical research. Currently though suitable alternatives are widely available, either in the form of open-access repositories such as the Open Science Framework (https://cos.io/our-products/open-science-framework) or Dataverse (https://dataverse.org/), or in the form of so-called “registered reports” format that is increasingly adopted by scientific journals, in which the study protocol is pre-registered and peer-reviewed before the experiment is conducted.[63,64] For more clinically oriented studies the ‘US National Institutes of Health Trial Register’ and ‘European Clinical Trial Register’ are respected platforms for registration.

#### 4.2.2. Selection, detection and performance bias

Other necessary methodological improvements to minimize the risk of selection, detection, and performance bias include a detailed assessment of participants’ baseline and background characteristics, proper blinding of personnel, and a manipulation check to ascertain the extent to which the experimental interventions indeed resulted in relatively more implicit/explicit motor learning.

First, participants’ baseline and background characteristics should be described in detail, to ascertain sufficient group comparability. Most importantly, participants should be tested before commencement of the intervention to assess whether the investigated groups are similar in terms of motor ability. It has been argued that such a baseline assessment test should not involve the exact same motor task as during the training- and test phase, because learners would already acquire explicit knowledge of the to-be-learned motor skill, and hence be less able to learn implicitly. In fact, this is why many researchers purposely have discarded the use of pretest assessments in implicit motor learning research.[31] One way to avoid this might be to have participants perform a baseline assessment on a different, presumably related motor task (e.g., measure participants’ sway during upright standing as baseline-test for stabilometer practice). This is only a preliminary suggestions of how baseline assessments may be done, whilst trying to prevent that subsequent implicit learning is thwarted. Future research is needed to test this approach, or to find alternative, possibly more suitable ideas to address this problem.

Second, with regard to blinding, future studies should strive to have independent and blinded researchers perform the group allocation and outcome assessment. This minimizes the possibility that the experimenter will be (subconsciously) influenced while performing allocation and pre- and post (retention)-tests. Blinding of the person who administers the intervention will be more difficult to achieve, if not impossible. One way to minimize such performance bias could be to appoint a research assistant with sufficient experimental skills (e.g., in the domains of biomechanics, physiology, social psychology), but who does not have in-depth knowledge of motor learning theories and is not aware of the research question and expected results. However, this will only partly reduce the performance bias risk; it cannot be ruled out that with time this person will figure out the hypothesis under investigation.

Third, studies must always include a manipulation check, in the form of an assessment of learners’ declarative movement-related knowledge after practice is terminated. It is preferable if such assessments also probe a learner’s episodic knowledge and not only the accumulated generic knowledge, as the former is more closely linked to the degree to which people explicitly control their performance.[65] A clear strength of the current literature is that most studies already incorporate episodic knowledge assessments,[13,30–32,39–50,52,58] Future studies could also screen episodic knowledge reports for hypothesis testing statements, which may be particularly indicative of explicit learning (e.g. [31]).

The above described suggestions illustrate how the robustness of research practices in the field of motor learning may be improved. They complement recommendations made by Lohse and co-workers[27] who additionally highlighted statistical biases in motor learning research. Combined, these suggestions may be suitable starting points for a so-called Delphi study in which motor learning experts along with statistical and methodological experts try to find consensus on standard protocols and reporting guidelines for different types of motor learning research.

### 4.3. Implications for research

There is some evidence that implicit motor learning improves movement automatization compared to explicit motor learning, but there is obviously a need to further strengthen the level of evidence. Based on our findings, analogy learning interventions may be best suited for further research, as it seemed most apt at inducing implicit motor learning. When we only consider the comparisons for which participants’ declarative knowledge reports were available, analogy learning interventions most consistently effectuated implicit motor learning. Specifically, analogy groups reported less declarative knowledge than the explicit groups in all 12 comparisons that performed such checks.[30,32,40–42,47,52,55,58] In contrast, comparisons that concerned errorless learning (7 out of 12 comparisons[13,39,44,47,51]) were considerably less successful in this regard. Results are unclear for external focus interventions; one comparison revealed less explicit knowledge for the implicit group,[50] two did not,[46,50] while declarative knowledge checks were unavailable for the remaining 5 comparisons.[15,40,56,57] Dual-task learning successfully induced implicit motor learning in the one experiment that we included[32] (see also Masters[1]).

Relatedly, retention intervals influenced whether interventions successfully elicited implicit- and explicit motor learning. Manipulation checks were more often positive for comparisons that concerned a delayed retention test (N = 6/6) than for comparisons that concerned immediate retention tests (N = 15/24). Thus, a sharper distinction between implicit- and explicit learning interventions may be achieved when the retention tests are delayed by at least one night’s sleep. This would be in line with findings that sleep results in better consolidation of both declarative and procedural knowledge (e.g. [66]), which may enhance the contrast between these knowledge types. Since the variety in used retention intervals could possibly affect the studies’ outcome, one should strive to a fixed retention interval of more than 24 hours.[28,29]

Also, we recommend that studies not only compare explicit and implicit groups’ motor DT performance, but also compare the extent to which performance deteriorates in DT compared to ST conditions–for instance by calculating DT costs.[35] In addition, the calculation of (cognitive) DT costs of the secondary task is required when examining the degree of movement automaticity. This could only be obtained from one[15] of the reviewed studies. Without cognitive DTC assessment it is impossible to say whether group differences in the primary motor DT performance and motor DTCs are not simply due to group differences in task prioritization during dual-tasking. Relatedly, it is important that researcher use task priority instructions and report these.

Further research may also validate potentially more objective methods than DT performance to assess the degree of movement automaticity. A promising addition is the use of EEG measurements. Zhu and co-workers found that increased movement automaticity is characterized by reduced coherence between left-sided verbal-analytical brain regions (T3-electrode) and central premotor brain regions (Fz).[67,68] In addition to this, there is evidence that task-irrelevant probes elicit less distinct event-related potentials (e.g., reduced amplitude of the P3 component observed at the Pz electrode) when the motor task is more automatic.[69]

Future research should also strive for longer practice periods. The majority of studies in this review only involved a single practice session.[13,31,32,40,43–53,55,57,58] As such, most of the evidence concerns the very early stages of learning, but relatively little is known about the long-term effects of implicit and explicit motor learning interventions (see Koedijker et al.,[41] Schücker et al.,[54] and Maxwell et al.[11] for noteworthy exceptions). There is good chance that differences in single- and dual-task motor performance become smaller with increased practice duration, given that–with sufficient practice–explicit learning should also lead to similar degrees of automaticity.[41] A sufficiently long practice period would allow researchers to compare movement automaticity between implicit and explicit groups at the end-stage, but also intermediate stages of skill development. This allows more fine-grained assessment of the degree to which implicit motor learning enhances movement automatization at different learning phases and/or skill levels.

Finally, future studies should incorporate larger samples, based on appropriate power calculations. Most studies in this review concerned relatively small groups (mean N = 14 per experimental group). If studies lack sufficient power they are less likely to find significant effects. Also, when they do find an effect it is more likely to over- or understate the “true” effect.[27,70]

### 4.4. Implications for practice

For sports practice, there is currently not sufficiently strong evidence for the superiority of implicit interventions over explicit ones–at least not when it comes to improving automaticity (and dual-tasking). Please note that we did not assess other possible benefits of implicit motor learning to athletic performance (i.e., greater single-task performance increase, more resilient performance in fatiguing and high-stress conditions), so it is certainly possible that implicit motor learning benefits performance in other ways. For now, it may therefore be best for coaches and trainers to incorporate both approaches in their practice regimes, sometimes encouraging their athletes to use a more explicit approach and sometimes stimulating them to learn relatively implicitly. Based on this review, analogy learning may be one of the most promising implicit learning methods for practical application, although it certainly does require some ingenuity on the part of the coach to find proper individualized and meaningful analogies for each athlete for different tasks. There is currently no direct evidence that tells us in what circumstances it is best to either opt for an implicit or explicit approach. Some have hypothesized that explicit learning is best suited for improving (strategic) action selection (i.e., which movement solution is best for the given situation), whereas implicit strategies may be more suitable to refine the actual implementation of the movement. Also, it has been postulated that explicit approaches are useful when athletes want to improve or refine a firmly consolidated yet “attenuated” motor skill, or when they are confronted with novel, complex situations.[71] Coaches may also want to take into account their athletes’ preferences and working memory; people with explicit motor control preferences and larger working memory capacity may benefit more from explicit interventions, and vice versa.[72,73] Still, please note that these are but hypotheses that await verification, and can by no means be used as fixed guidelines.

### 4.5. Strengths and limitations

We used a highly sensitive search strategy that was formulated by a research librarian and motor learning expert, and that encompassed numerous conventional electronic databases, grey literature sources, trial registers and hand searching of reference lists of included studies. Another strength is that all steps in the review process were performed by two independent reviewers. In addition, an epidemiologist and motor learning expert independently thoroughly assessed studies’ risk of bias by means of the reference standard, the Cochrane’s risk of bias tool. Nonetheless, several limitations remain.

First, this review was specifically restricted to the question of whether implicit motor learning leads to a greater degree of automatization of sports-related motor tasks compared to explicit motor learning. By doing so, we only focused on dual-task performance. Although single-task performance was assessed as well, this was only done for those studies that also looked at DT performance. Therefore, the fact that a few studies showed a benefit of implicit learning over explicit learning for single-task performance may be taken as indicative for the larger number of studies available on this topic, they are by no means definitive. Also, this review did not look into other presumed benefits of implicit motor learning, such as more robust performance under psychological and physiological stress (e.g. [1]). However, as these benefits are assumed to be associated with implicit motor learning resulting in accelerated movement automation, it was deemed to be most important to first scrutinize this latter proposition.

A second limitation is that for this review we relied on learners’ self-reported declarative knowledge to verify whether the explicit group indeed learned more explicitly than the implicit group. There is debate regarding the validity of this approach, as it is unclear whether the rules reported post-learning are also actually used during practice, and whether their use actually gave rise to the observed performance improvements. Also, this measure may not be particularly sensitive to detect group differences.[74] Still, despite their limitations, verbal reports are currently the best available and most frequently used measures, that can best be compared across studies.

A third limitation is the absence of data synthesis by means of meta-analysis. Such an analysis allows to weigh studies according to their relative sample sizes and/or the precision of the effect estimate, and would therefore have provided more detailed insight into the relative effectiveness of implicit and explicit motor learning. However, the unclear risk of bias compromised the validity of meta-analysis, and required us to limit ourselves to a descriptive data synthesis.[36]

Fourth, the review was limited to four types of implicit motor learning interventions, namely analogy learning, errorless learning, dual-task learning, and external focus learning. This approach resulted in the exclusion of several other used interventions, such as discovery learning, which narrows the scope of this review. Also, while experts and practitioners generally agreed upon the former three interventions to be implicit motor learning interventions,[10] they did not label external focus learning as such. Nonetheless, this intervention was incorporated in this review, because there are indications that external focus learning is a relatively implicit form of learning,[12] that is suggested to result in a reduced build-up of declarative knowledge.[33,50]

Finally, the presence of publication bias was assessed by means of a funnel plot. However, not all comparisons could be included, due to missing standard deviations for certain comparisons. Hence, the possibility of publication bias cannot be completely excluded.

### 4.6. Conclusions

This study found level 3 evidence for a small positive effect of implicit motor learning on movement automaticity when compared with explicit motor learning. There is a clear need to further investigate the possible benefits of implicit motor learning for sports practice. This calls for uniform, motor learning-specific guidelines on design and reporting, to enable low-risk-of-bias trials that yield stronger evidence.

## 5. Acknowledgments

We would like to thank Ralph de Vries of the medical library of the Vrije Universiteit Amsterdam for his help with optimizing our search strategy.

## Supporting information

S1 Text Search strategy. Example of the search strategy for Medline.
(DOCX)

S1 Fig Funnel plot of included studies. NB: Only comparisons for which standard deviations were available could be included in the funnel plot. Assessment was conducted on the difference in absolute motor dual-task performance (X-axis) between implicit and explicit groups at the latest reported test-phase; experiments with positive value on X-axis indicate better dual-task performance for the implicit group, in contrast to negative values which suggest explicit superiority. The secondary task involved, was exclusively cognitive (e.g. tone judgement, counting). Only Orrell et al.[47,48] executed a secondary motor task, which was not entered in this analysis. Some experiments consisted of more than one test phase[41,48,53] or motor outcome[54]. Therefore, multiple funnel plots were conducted to inspect whether this affected the result, but this was not the case.
(PDF)

S1 Table Cochrane risk of bias tool. (PDF)

S2 Table Study characteristics. (PDF)

S3 Table Prisma checklist. (PDF)

S1 File Study data. (RM5)
