# Supplementary material for: Does implicit motor learning lead to greater automatization of motor skills compared to explicit motor learning? A systematic review
Source: PLoS One. 2018 Sep 5;13(9):e0203591. doi: 10.1371/journal.pone.0203591 (PMC6124806; doi:10.1371/journal.pone.0203591)
Supplement: S1 Text — Example of the search strategy for Medline. (DOCX) [file pone.0203591.s001.docx]

**S1 Text. Search Strategy**. Example of the search strategy for Medline.

| **#1** | "Learning"[Mesh] OR Learn*[tiab] OR memor*[tiab] OR knowledge[tiab] |
| --- | --- |
| **#2** | Implicit*[tiab] OR procedural*[tiab] OR unintentional*[tiab] OR incidental*[tiab] OR nondeclarative[tiab] OR non declarative[tiab] OR analogy[tiab] OR analogies[tiab] OR errorless[tiab] OR dual task[tiab] OR external*[tiab] OR observational*[tiab] OR unconscious*[tiab] OR Explicit*[tiab] OR internal*[tiab] OR reinvestment*[tiab] OR discover*[tiab] OR trial and error*[tiab] OR declarative*[tiab] OR conscious*[tiab] |
| **#3** | "Psychomotor Performance"[Mesh] OR "Motor skills"[Mesh] OR psychomotor*[tiab] OR task perform*[tiab] OR motor*[tiab] OR movement*[tiab] OR muscle control*[tiab] OR muscular control*[tiab] |
| **#4** | "Sports"[Mesh] OR sport[tiab] OR sports[tiab] OR distract*[tiab] OR multi task*[tiab] OR ((dual[tiab] OR secondar*[tiab] OR concurrent*[tiab]) AND task*[tiab]) OR (cognitive*[tiab] AND demand*[tiab]) OR (attention*[tiab] AND demand*[tiab]) |
| **#5** | (#1 AND #2 AND #3 AND #4) NOT ("Animals"[Mesh] NOT "Humans"[Mesh]) |

**Hits: 1702 (02-03-2017)**
