# Supplementary figures and images for: Does implicit motor learning lead to greater automatization of motor skills compared to explicit motor learning? A systematic review
Source: PLoS One. 2018 Sep 5;13(9):e0203591. doi: 10.1371/journal.pone.0203591 (PMC6124806; doi:10.1371/journal.pone.0203591)

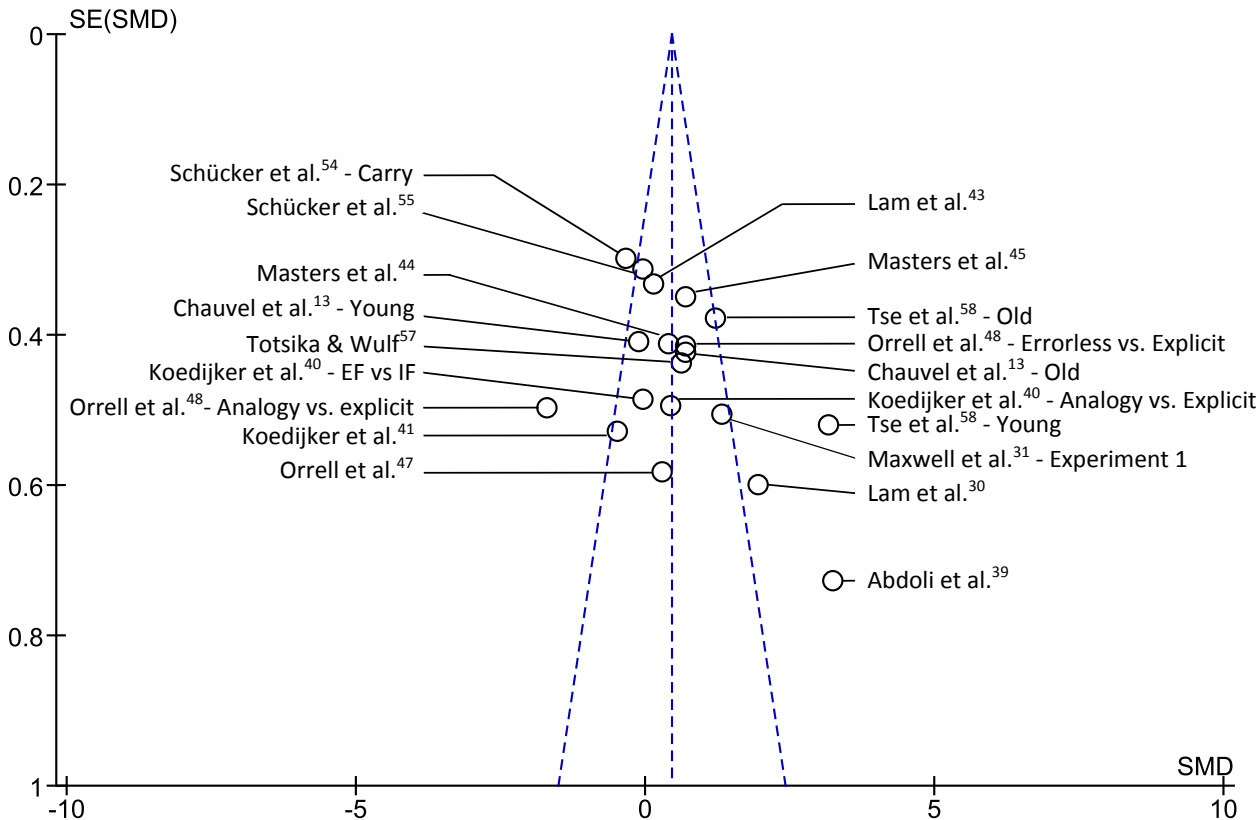

Supplement: S1 Fig — NB: Only comparisons for which standard deviations were available could be included in the funnel plot. Assessment was conducted on the difference in absolute motor dual-task performance (X-axis) between implicit and explicit groups at the latest reported test-phase; experiments with positive value on X-axis indicate better dual-task performance for the implicit group, in contrast to negative values which suggest explicit superiority. The secondary task involved, was exclusively cognitive (e.g. tone judgement, counting). Only Orrell et al.[47,48] executed a secondary motor task, which was not entered in this analysis. Some experiments consisted of more than one test phase[41,48,53] or motor outcome[54]. Therefore, multiple funnel plots were conducted to inspect whether this affected the result, but this was not the case. (PDF) [file pone.0203591.s002.pdf]
