# Supplementary material for: Does implicit motor learning lead to greater automatization of motor skills compared to explicit motor learning? A systematic review
Source: PLoS One. 2018 Sep 5;13(9):e0203591. doi: 10.1371/journal.pone.0203591 (PMC6124806; doi:10.1371/journal.pone.0203591)
Supplement: S1 Table — (PDF) [file pone.0203591.s003.pdf]

S1 Table. Cochrane Risk of Bias Tool

Domain of bias	*Qualification*	*Criteria for assigning risk of bias*
Selection bias		*Sequence generation*
	+	Computer based random number generators, a table with random numbers or similar methods
	-	Quasi randomization procedures e.g. allocation based on date of birth or on day of the week
	?	None described sequence generation
Selection bias		*Allocation concealment*
	+	Person(s) responsible for randomization should be independent and blinded to participant at randomization
	-	Person(s) responsible for randomization should are not independent or blinded to participant at randomization
	?	None described allocation concealment
Performance bias (1)		*Blinding of participants and personnel*
	+	Blinded participants and personnel;<br>*It is stated that participants were not specifically informed about the nature of the intervention*<br>*Personnel that provided intervention was not informed about nature of the intervention*
	-	Non blinded participants and personnel;<br>*It is not stated that participants were not specifically informed about the nature of the intervention*<br>*Personnel that provided intervention was not informed about nature of the intervention*
	?	None described or unclear blinding of participants and personnel
Performance bias (2)		*Manipulation check of degree to which motor learning had been implicit/explicit*
	+	Implicit group demonstrated significantly less movement-related knowledge than explicit group after learning
	-	No clear differences in movement-related knowledge between implicit and explicit groups
	?	No manipulation checks described/reported
Detection bias		*Blinding of outcome assessment*
	+	Blinded outcome assessor
	-	Non blinded outcome assessment
	?	Methods of (blinding) the outcome assessment were not described
Attrition bias		*Incomplete outcome data*
	+	Random lost to follow up of participants was present when ≤ 10% was lost to follow up
	-	Selective lost to follow up of participants was present when > 10% was lost to follow up
	?	Unclear lost to follow up
Reporting bias		*Selective reporting* (www.controlled-trials.com, ClinicalTrials.gov, http://apps.who.int/trialsearch/ were searched for protocols)
	+	Articles that reported all a priori described outcomes
	-	Articles that did not report all a priori described outcomes
	?	The protocol was not found.
Other biases		
	+	No other systematic errors were present
	-	Any other systematic errors that could lead to bias (e.g., baseline differences between groups in motor skill, or other possibly relevant factors)
