# Supplementary material for: Does implicit motor learning lead to greater automatization of motor skills compared to explicit motor learning? A systematic review
Source: PLoS One. 2018 Sep 5;13(9):e0203591. doi: 10.1371/journal.pone.0203591 (PMC6124806; doi:10.1371/journal.pone.0203591)
Supplement: S2 Table — (PDF) [file pone.0203591.s004.pdf]

## S2 Table. Study Characteristics.

| Study | Participants | Task | Intervention | Outcome measures | Results (retention) |
|---|---|---|---|---|---|
| Abdoli et al. (2012) [39] | **Number at baseline:** 30<br><br>**Inclusion/exclusion criteria:**<br>- No visual/motor impairment<br><br>**Number of groups: 3**<br>- Errorless (n=10)<br>- Errorful (n=10)<br>- Control (n=10)<br><br>**General descriptives:**<br>- Gender (m/f): 0/30<br>- Age (years): 22±2.0<br>- All participants were right handed<br><br>**Specific group characteristics:** N/A<br><br>**Pre-test single task motor performance:** N/A | Ball-throwing task<br>- Hitting a concentric target on a board by bouncing the ball via a specific target zone on the ground<br>- Maximum score: 10 points/throw | **Groups of interest:**<br>- Errorless ('Implicit'): Distance from target was progressively increased (2.5-3.5 m in 0.25 m steps).<br>- Errorful ('Explicit'): Distance from target was progressively reduced (3.5-2.5 m in 0.25 m steps);<br><br>**Procedure:**<br>*Days 1-3: Learning phase:*<br>- 5 blocks 25 ST trials / day<br>- End of day 3: 1 block of 10 ST trials at 3 m distance<br>*Day 4:Test Phase:*<br>- 2 test blocks of 10 trials, at 3 m distance:<br>  ○ ST<br>  ○ DT (counting backwards from 1100 in steps of 3)<br>- 2 transfer tests (throwing from shorter/longer distance than 3 m) | ***Primary outcome:***<br>- Primary motor task: Throwing precision (number of points awarded per block; M± SD)<br>- Secondary task: Counting accuracy & speed (M±SD)<br><br>***Secondary outcome:***<br>- Declarative knowledge: Number of explicit rules (M±SD) | **Motor task performance:**<br>*Implicit*<br>- ST = 49.4±4.5<br>- DT = 46.3±5.3<br>- DTC= 6.3%<br>*Explicit*<br>- ST = 41.9±5.3<br>- DT = 31.2±3.4<br>- DTC = 25.5%<br><br>**Secondary task performance:**<br>*Implicit*<br>- ST = N/A<br>- DT<br>  ○ Accuracy = 87±77%<br>  ○ Speed = 13.68±2.97<br>- DTC = N/A<br>*Explicit*<br>- ST = N/A<br>- DT<br>  ○ Accuracy = 66±13%<br>  ○ Speed = 10.99±5.63<br>- DTC = N/A<br><br>**Declarative knowledge:**<br>*Implicit* = 5.1±1.0<br>*Explicit* = 9.4±1.8<br><br>**Implicit versus Explicit comparison:**<br>- Motor ST: $p$=.003<br>- Motor DT: $p$<.0001<br>- Motor DTC: $p$<.05<br>- Secondary DT:<br>  ○ Accuracy: $p$=.41<br>  ○ Speed: $p$=.20<br>- Secondary DTC: N/A |

| | | | | | |
|---|---|---|---|---|---|
| | | | | | - Declarative knowledge: *p*<.0001 |
| Chauvel et al. (2012) [13] | **Number at baseline:**<br>- 48 younger adults<br>- 48 older adults<br><br>**Inclusion/exclusion criteria:**<br>- No visual/hearing impairments<br>- No history of neurological disease<br>- No medication that affects cognition.<br><br>**Number of groups: 4**<br>- Errorful-young (n=24)<br>- Errorless-young (n=24)<br>- Errorful-old (n=24)<br>- Errorless-old (n=24)<br><br>**General descriptives:**<br>- Younger adults<br>o Gender (m/f): 24/24<br>o Age (years): 23.5±3.3<br>- Older adults<br>o Gender (m/f): 25/23<br>o Age (years): 65.0±3.7<br><br>**Specific group characteristics:** N/A<br><br>**Pre-test single task motor performance:** N/A | Golf putting task | **Groups of interest:**<br>- Errorless ('Implicit'): Distance from target was progressively increased (0.25-1.0 m in 0.25 m steps)<br>- Errorful ('Explicit'): Distance from target was progressively reduced (2.25-1.5 m in 0.25 m steps)<br><br>**Procedure:**<br>*Day 1: Learning phase:*<br>- 4 blocks of 40 ST trials.<br>*Day 1: Test phase:*<br>- One 40 trial-block, at 1.25 m distance<br>- Groups were split:<br>o ST: ½ of errorless & errorful groups<br>o DT: ½ of errorless & errorful groups (secondary tone counting while putting) | ***Primary outcome:***<br>- Primary motor task: Number of successful putts (M±SD)<br>- Secondary task: Counting accuracy (%; M±SD)<br><br>***Secondary outcome:***<br>- Declarative knowledge: Number of explicit hypothesis (M±SD) | **Motor task performance:**<br>*Implicit-young*<br>- ST = 29.1±7.0<br>- DT = 26.9±5.0<br>- DTC = N/A<br>*Explicit-young*<br>- ST = 29.1±3.0<br>- DT = 27.5±4.3<br>- DTC = N/A<br>*Implicit-old*<br>- ST = 24.1±10.7<br>- DT = 26.5±7.0<br>- DTC = N/A<br>*Explicit-old*<br>- ST = 24.0±6.7<br>- DT = 21.5±6.7<br>- DTC = N/A<br><br>**Secondary task performance:**<br>*Implicit-young*<br>- ST = N/A<br>- DT = 87.5±14.1%<br>- DTC = N/A<br>*Explicit-young*<br>- ST = N/A<br>- DT = 89.2±8.8%<br>- DTC = N/A<br>*Implicit-old*<br>- ST = N/A<br>- DT = 85.7±9.2%<br>- DTC = N/A<br>*Explicit-old*<br>- ST = N/A<br>- DT = 78.6±9.9%<br>- DTC = N/A<br><br>**Declarative knowledge:**<br>*Implicit – young&old combined:*<br>1.2 ± 0.9 |

| | | | | | |
|---|---|---|---|---|---|
| | | | | | *Explicit – young&old combined:*<br>1.6 ± 1.0<br><br>**Implicit versus Explicit comparison:**<br>*Young groups*<br>- Motor ST: *p*=1.0<br>- Motor DT: *p*=.76<br>- Motor DTC: N/A<br>- Secondary DT: *p*=.73<br>- Secondary DTC: N/A<br>*Old groups*<br>- Motor ST: *p*=.96<br>- Motor DT: *p*=.09<br>- Motor DTC: N/A<br>- Secondary DT: *p*=.08<br>- Secondary DTC: N/A<br>*Combined groups:*<br>- Declarative knowledge: *p*=.042 |
| Koedijker et al. (2007) [40] - ***Analogy vs. Explicit*** | **Number at baseline:**<br>17<br><br>**Inclusion/exclusion criteria:**<br>- No experience in table tennis.<br><br>**Number of groups: 2**<br>- Explicit (n=9)<br>- Analogy (n=8)<br><br>**General descriptives** *(of total sample – combined with Koedijker et al., 2007 – External vs. Internal focus):*<br>- Gender (m/f): 7/27<br>- Age (years): 21.8±3.6<br><br>**Specific group characteristics:** N/A<br><br>**Pre-test single task motor performance:**<br>- Explicit = 1.63±0.59 | Table tennis task<br>- Participants stood opposite a ball machine and hit the ball to target at opposite side of table | **Groups of interest:**<br>- Analogy ('Implicit'): "Pretend drawing a right-angled triangle with the bat"<br>- Explicit ('Explicit'): Explicit instructions on table tennis forehand performance<br><br>**Procedure**<br>*Day 1: Pretest:*<br>- 50 ST trials<br>*Day 1: Learning phase*<br>- 9 blocks of 50 ST trials<br>*Day 1: Test phase:*<br>- 3 test blocks of 50 trials:<br>  o Low-pressure ST (LPT-ST)<br>  o High-pressure ST (HPT-ST)<br>  o Low-pressure DT (counting backwards from 1100 in steps of 3) | ***Primary outcome****:*<br>- Primary motor task: Combined score of accuracy and movement execution (M± SD)<br>- Secondary task: Counting accuracy (%) and speed (s; M± SD)<br><br>***Secondary outcome:***<br>- Declarative knowledge: Number of explicit (combined internal and external focus) rules (M± SD) | **Motor task performance:**<br>*Implicit*<br>- (LPT-)ST = 2.88±1.21<br>- DT = 3.09±1.66<br>- DTC= -7.3%<br>*Explicit*<br>- (LPT-)ST = 3.23±0.87<br>- DT = 2.43±1.18<br>- DTC= 24.8%<br><br>**Secondary task performance:**<br>*Both groups combined:*<br>- ST = N/A<br>- DT =<br>  o Accuracy: 96-98%<br>  o Speed: 2.46-2.87 s<br><br>**Declarative knowledge:**<br>*Implicit*: 4.0±1.8<br>*Explicit:* 7.6±2.8<br><br>**Implicit versus Explicit comparison:**<br>- Motor ST: *p*=.50 |

| | | | | | |
|---|---|---|---|---|---|
| | - Analogy = 1.08±0.45<br>- T-test on pretest: *p*=.049 | | | | - Motor DT: *p*=.37<br>- Motor DTC: *p*>.05<br>- Secondary DT:<br>○ Accuracy: *p*>.05<br>○ Speed: *p*>.05<br>- Secondary DTCs: N/A<br>- Declarative knowledge: *p*=.007 |
| Koedijker et al. (2007) [40] – ***External vs. Internal Focus*** | **Number at baseline:**<br>17<br><br>**Inclusion/exclusion criteria:**<br>- Same as described above (Koedijker et al. 2007 – Analogy vs. Explicit Learning)<br><br>**Number of groups: 2**<br>- External Focus (n=9)<br>- Internal Focus (n=8)<br><br>**General descriptives:**<br>Same as described above (Koedijker et al. 2007 – Analogy vs. Explicit Learning)<br><br>**Specific group characteristics:** N/A<br><br>**Pre-test single task motor performance:**<br>- External = 1.49±0.76<br>- Internal = 1.66±1.00<br>- T-test on pretest: *p*=.70 | Table tennis task<br>- Same as for Koedijker et al. 2007 – Analogy vs. Explicit Learning | **Groups of interest:**<br>- External focus ('Implicit'): "Attend to the ball at all times"<br>- Internal focus ('Explicit'): "Focus on movement execution"<br><br>**Procedure**<br>Identical to Koedijker et al. 2007 – Analogy vs. Explicit Learning | ***Primary outcome:***<br>- Primary motor task: Combined score of accuracy and movement execution (M± SD)<br>- Secondary task: Counting accuracy (%) and speed (s; M± SD)<br><br>***Secondary outcome:***<br>- Declarative knowledge: Number of explicit (combined internal and external focus) rules (M±SD) | **Motor task performance:**<br>*Implicit*<br>- (LPT-)ST = 2.53±1.01<br>- DT = 2.18±0.92<br>- DTC= 13.8%<br>*Explicit*<br>- (LPT-)ST = 2.60±1.00<br>- DT = 2.22±1.01<br>- DTC= 14.6%<br><br>**Secondary task performance:**<br>*Both groups combined:*<br>- ST = N/A<br>- DT =<br>○ Accuracy: 96-98%<br>○ Speed: 2.46-2.87 s<br><br>**Declarative knowledge:**<br>*Implicit*: 6.5±3.1<br>*Explicit:* 4.9±2.0<br><br>**Implicit versus Explicit comparison:**<br>- Motor ST: *p*=.89<br>- Motor DT: *p*=.93<br>- Motor DTC: *p*>.05<br>- Secondary DT: *p*>.05<br>- Secondary DTC: N/A<br>- Declarative knowledge: *p*=.23 |
| Koedijker et al. (2008) [41] | **Number at baseline:**<br>15<br><br>**Inclusion/exclusion criteria:**<br>- No experience in table tennis | Table tennis task<br>- Participants stood opposite a ball machine and hit the ball to target at | **Groups of interest:**<br>- Analogy ('Implicit'): "Pretend drawing a right-angled triangle with the bat"<br>- Explicit ('Explicit'): Explicit instructions on table tennis forehand performance | ***Primary outcome:***<br>- Primary motor task: Combined score of accuracy and movement execution (M±SD)<br>- Secondary task: Number of incorrect | **Motor task performance:**<br>***Test phase 1 (Immediate)***<br>*Implicit*<br>- (LPT-)ST = 4.67±2.35<br>- DT = 4.47±2.31<br>- DTC= 4.3%<br>*Explicit* |

| | | | | | |
|---|---|---|---|---|---|
| | **Number of groups: 2**<br>- Analogy (*n*=7)<br>- Explicit (*n*=8)<br><br>**General descriptives:**<br>- Gender (m/f): 2/13<br>- Age (years): 19.6±3.4<br><br>**Specific group characteristics:** N/A<br><br>**Pre-test single task motor performance:**<br>- Analogy = 1.94±1.48<br>- Explicit = 1.33±0.73<br>- T-test on pretest: *p*=.32 | opposite side of table | **Procedure:**<br>6 sessions, spread over six weeks:<br>*Week 1: Pretest (PT):*<br>- 50 ST trials<br>*Week 1: Early learning phase:*<br>- 14 blocks of 100 ST trials<br>*Week 1: Test phase 1:*<br>- 3 test blocks of 50 trials<br>o Low-pressure ST (LPT-ST)<br>o High-pressure ST (HPT-ST)<br>o DT (counting backwards from 1100 in steps of 3)<br>*Weeks 2 -5: Prolonged learning phase:*<br>- Sessions 2&3: 15 blocks of 100 trials<br>- Sessions 4&5: 20 blocks of 100 trials<br>*Week 6: Test phase 2:*<br>- Procedure similar to test phase 1, except that there was no pretest. | counts, and number of counts/second (M±SD)<br><br>***Secondary outcome:***<br>- Declarative knowledge: Number of explicit (combined internal and external focus) rules (M± SD) | - (LPT-)ST = 4.09±1.60<br>- DT= 3.84±1.87<br>- DTC= 6.1%<br>***Test phase 2 (Delayed)***<br>*Implicit*<br>- (LPT-)ST = 4.42±1.53<br>- DT = 4.24±1.06<br>- DTC= 4.1%<br>*Explicit*<br>- (LPT-)ST = 5.56±0.95<br>- DT= 4.84±1.21<br>- DTC= 12.9%<br><br>**Secondary task performance:**<br>*Both groups and test phases combined:*<br>- ST = N/A<br>- DT =<br>o Accuracy: 2.8±2.5 incorrect<br>o Speed: 0.5/s<br><br>**Declarative knowledge:**<br>***Test phase 1 (Immediate)***<br>*Implicit*: 2.9±1.7<br>*Explicit:* 7.5±2.4<br>***Test phase 2 (Delayed)***<br>*Implicit*: 2.4±0.6<br>*Explicit:* 5.0±1.8<br><br>**Implicit versus Explicit comparison:**<br>***Test phase 1 (Immediate)***<br>- Motor ST: *p*=.58<br>- Motor DT: *p*=.57<br>- Motor DTC: *p*>.31<br>- Secondary DT: *p*>.39<br>- Secondary DTC: N/A<br>- Declarative knowledge: *p*=.001<br>**Test phase 2 (Delayed)**<br>- Motor ST: *p*=.10<br>- Motor DT: *p*=.33<br>- Motor DTC: *p*>.31<br>- Secondary DT: *p*>.39 |

| | | | | | |
|---|---|---|---|---|---|
| | | | | | - Secondary DTC: N/A<br>- Declarative knowledge: *p*=.003 |
| Lam et al.(2009a) [42] | **Number at baseline:**<br>24<br><br>**Inclusion/exclusion criteria:**<br>- Only female participants<br><br>**Number of groups: 2**<br>- Analogy (n=12)<br>- Explicit (n=12)<br><br>**Specific group characteristics:**<br>- Analogy:<br>○ Gender (m/f): 0/12<br>○ Age (years): 21.9±1.7<br>○ Weight (kg): 53.8±9.7<br>○ Height (m): 1.61±0.04<br>- Explicit:<br>○ Gender (m/f): 0/12<br>○ Age (years): 21.1±1.2<br>○ Weight (kg): 53.9±6.5<br>○ Height (m): 1.63±0.04<br><br>**Pre-test single task motor performance: N/A**<br><br>**Pre-test single task secondary task performance:**<br>- Analogy: 452.3±79.7<br>- Explicit: 424.3±67.9 | Basketball free throws<br>- Shooting from a seated position into a standard basketball rim | **Groups of interest:**<br>- Analogy (‘Implicit’):<br>“Shoot as if you are trying to put cookies into a cookie jar on a high shelf.”<br>- Explicit (‘Explicit’):<br>Eight instructions describing correct shooting technique<br><br>**Procedure:**<br>*Day 1&2: Learning phase:*<br>- 6 blocks of 40 trials<br>- All participants aimed to maximize their shooting performance while responding as quickly as possible to auditory probes<br>*Day 3:Test phase:*<br>- 2 retention blocks of 40 trials; ad random trials were performed<br>○ Without auditory probes<br>○ With auditory probes presented before movement initiation<br>○ With auditory probes presented during movement execution<br>- 1 transfer block (similar procedure as for retention, but in high-pressure conditions) | ***Primary outcome:***<br>- Primary motor task:<br>Accuracy (number of points per block; M± SD)<br>- Secondary task:<br>Probe reaction time (ms; M±SD)<br><br>***Secondary outcome:***<br>- Declarative knowledge:<br>Number of explicit rules (M±SD) | **Motor task performance: ?**<br>*Authors do not report motor performance for trials with and trials without probes for implicit and explicit groups separately*<br><br>**Secondary task performance:**<br>*Implicit*<br>- ST = N/A<br>- DT (execution)= 447.7 ms<br>*Explicit*<br>- ST = N/A<br>- DT (execution)= 455.7 ms<br><br>**Declarative knowledge:**<br>*Implicit*= 1.9±1.3<br>*Explicit* = 6.2±2.2<br><br>**Implicit versus Explicit comparison:**<br>- Motor ST: *p*>.10<br>- Motor DT: *p*>.10<br>- Motor DTC: *p*>.05<br>- Secondary DT: *p*>.05<br>- Secondary DTC: N/A<br>- Declarative knowledge: *p*<.0001 |
| Lam et al. (2009b) [30] | **Number at baseline:**<br>27<br><br>**Inclusion/exclusion criteria:**<br>- No previous experience with basketball shooting<br><br>**Number of groups: 3**<br>- Explicit (n=9)<br>- Analogy (n=9) | Basketball free throws<br>- Shooting from a seated position into a standard basketball rim. | **Groups of interest:**<br>- Analogy (‘Implicit’):<br>“Shoot as if you are trying to put cookies into a cookie jar on a high shelf.”<br>- Explicit (‘Explicit’):<br>8 instructions describing correct shooting technique<br><br>**Procedure:** | ***Primary outcome:***<br>Primary motor task:<br>Accuracy (number of points per block; M±SD)<br>- Secondary task:<br>Counting accuracy (%) and speed (counts/minute; M±SD)<br><br>***Secondary outcome:*** | **Motor task performance:**<br>*Implicit*<br>- ST = 65.7±3.5<br>- DT = 65.7±4.1<br>- DTC= 0%<br>*Explicit*<br>- ST = 65.8±5.1<br>- DT = 59.3±1.6<br>- DTC= 9.9% |

| | | | | | |
|---|---|---|---|---|---|
| | - Control (n=9)<br><br>**General descriptives:**<br>- Gender (m/f): ?<br>- Age (years): 21.0±1.1<br>- Weight (kg): 49.1±5.7<br>- Height (m): 1.59±0.05<br><br>**Specific group characteristics: N/A**<br><br>**Pre-test single task motor performance: N/A** | | *Day 1-3: Learning phase:*<br>- 8 blocks of 20 ST trials per day<br>*Day 4:Test phase:*<br>- 3 test blocks of 20 trials:<br>o 2 ST blocks<br>o 1 DT blocks (counting aloud backward in threes from 1100) | - Declarative knowledge: Number of explicit rules (M±SD) | **Secondary task performance:**<br>*Implicit*<br>- ST = N/A<br>- DT<br>o Accuracy= 91.9%<br>o Speed= 20.8 counts/min<br>- DTC = N/A<br>*Explicit*<br>- ST = N/A<br>- DT<br>o Accuracy= 91.1%<br>o Speed= 21.1 counts/min<br>- DTC = N/A<br>-<br>**Declarative knowledge:**<br>*Implicit* = 2.7±0.8<br>*Explicit* = 7.9±2.2<br><br>**Implicit versus Explicit comparison:**<br>- Motor ST: $p$=.96<br>- Motor DT: $p$<.001<br>- Motor DTC: $p$<.05<br>- Secondary DT:<br>o Accuracy= $p$>.05<br>o Speed= $p$>.05<br>Secondary DTCs: ?<br>- Declarative knowledge: $p$<.0001 |
| Lam et al. (2010) [43] | **Number at baseline: 36**<br><br>**Inclusion/exclusion criteria:**<br>- No experience with golf putting or field hockey<br><br>**Number of groups:** 2<br>- Errorless (n=18)<br>- Errorful (n=18)<br><br>**General descriptives:**<br>- Gender (m/f): 22/14<br>- Age (years): 21.5±2.0 | Golf putting task | **Groups of interest:**<br>- Errorless ('Implicit'): Distance from target was progressively increased (0.25-2.0 m in 0.25 m steps)<br>- Errorful ('Explicit'): Distance from target was progressively reduced (2.0-0.25 m in 0.25 m steps)<br><br>**Procedure:**<br>*Day 1: Learning phase:*<br>- 8 blocks of 50 trials<br>- Ad random trials were performed<br>o Without auditory probes<br>o With auditory probes presented | ***Primary outcome:***<br>- Motor task: Accuracy (number of points per block; M±SD)<br>- Secondary task: Probe reaction time (ms; M±SD)<br><br>***Secondary outcome:***<br>- Declarative knowledge: Number of explicit (mechanical+hypothesis testing) rules (M± SD) | **Motor task performance:**<br>*Implicit*<br>- ST (no probe) = 101.1±38.6<br>- DT (execution) = 94.9±37.0<br>- DTC= 6.1%<br>*Explicit*<br>- ST (no probe) = 88.6±38.5<br>- DT (execution) = 88.8±35.4<br>- DTC= 0.2%<br><br>**Secondary task performance:**<br>*Implicit*<br>- ST = N/A<br>- DT = 513.7±100.6 |

| | | | | | |
|---|---|---|---|---|---|
| | **Specific group characteristics:** N/A<br><br>**Pre-test single task motor performance:** N/A | | before movement initiation<br>o With auditory probes presented during movement execution<br>*Day 1: Test phase:*<br>- 4 blocks of 50 trials, at 2 m distance:<br>o 2 blocks with similar procedure to practice blocks<br>o 2 transfer blocks: putting with unusual putters | | *Explicit*<br>- ST = N/A<br>- DT = 559.3±117.9<br><br>**Declarative knowledge:**<br>*Implicit* = 4.3±2.2<br>*Explicit* = 4.2±2.2<br><br>**Implicit versus Explicit comparison:**<br>- Motor ST: *p*=.34<br>- Motor DT: *p*=.73<br>- Motor DTC: *p*>.12<br>- Secondary DT: *p*=.39<br>- Secondary DTC: N/A<br>- Declarative knowledge: *p*=1.0 |
| Liao et al. (2001) [32]<br>***Experiment 1*** | **Number at baseline: 30**<br><br>**Inclusion/exclusion criteria:**<br>- Novice table tennis players<br>- Never received any form of instruction<br>- Never practiced more than once a fortnight<br><br>**Number of groups:** 3<br>- Analogy (n=10)<br>- Dual-task (n=10)<br>- Explicit (n=10)<br><br>**General descriptives:**<br>- Gender (m/f): 6/24<br>- Age (years): 27.5±4.4<br><br>**Specific group characteristics:**<br>- For each group, gender (m/f): 2/8<br><br>**Pre-test single task motor performance:** N/A | Table tennis task<br>- Hit table tennis ball onto target area with topspin using forehand stroke | **Groups of interest:**<br>- Analogy ('Implicit'): "pretend to draw a right-angled triangle with the bat"<br>- Dual-task ('Implicit'): Performed concurrent secondary task (random letter generation)<br>- Explicit ('Explicit'): Received 12 basic instructions on how to hit topspin forehand<br><br>**Procedure:**<br>*Day 1: Learning phase:*<br>- 6 blocks of 50 trials<br>*Day 1: Test phase:*<br>- 2 test blocks of 50 trials:<br>o ST<br>o DT (counting aloud backwards in threes from 1100) | ***Primary outcome:***<br>- Primary motor task: Accuracy (number of points per block; M±SD)<br>- Secondary task: Performance not assessed<br><br>***Secondary outcome:***<br>- Declarative knowledge: Number of explicit rules (M± SD) | **Motor task performance:**<br>*Implicit (analogy)*<br>- ST = 30.67<br>- DT = 29.92<br>- DTC= 2.4%<br>*Implicit (dual-task)*<br>- ST = 28.01<br>- DT = 27.46<br>- DTC= 2.0%<br>*Explicit*<br>- ST= 32.59<br>- DT = 18.81<br>- DTC= 42.3%<br><br>**Secondary task performance: N/A**<br>**Declarative knowledge:**<br>*Implicit (analogy)* = 1.51<br>*Implicit (dual-task)* = 0.97<br>*Explicit* = 6.54<br><br>**Implicit versus Explicit comparison:**<br>- Motor ST: ?<br>- Motor DT: ?<br>- Motor DTC:<br>o Analogy&Dual-Task groups |

| | | | | | |
|---|---|---|---|---|---|
| | | | | | vs. Explicit group: *p*<.01<br>○ Analogy vs. Dual-task group: *p*=.97<br>- Secondary DT: N/A<br>- Secondary DTC: N/A<br>- Declarative knowledge:<br>○ Analogy&Dual-Task vs. Explicit group: *p*<.001<br>○ Analogy vs. Dual-task group: *p*=.52 |
| Masters et al. (2008a) [44] | **Number at baseline: 36**<br><br>**Inclusion/exclusion criteria:**<br>- Right-handed<br>- No previous operative experience<br><br>**Number of groups:** 3<br>- Errorless (n=12)<br>- Explicit (n=12)<br>- Control (n=12)<br><br>**General descriptives:**<br>- Gender (m/f): 17/19<br>- Age (years): 22±3<br><br>**Specific group characteristics:** N/A<br><br>**Pre-test single task motor performance:** N/A | Suturing and knot tying task | **Groups of interest:**<br>- Errorless ('Implicit'): The correct position of suture points were pre-marked to reduce mistakes<br>- Explicit ('Explicit'): Detailed verbal instructions about the task were provided concurrent with demonstration of skill<br><br>**Procedure:**<br>*Day 1: Learning phase:*<br>- 5 familiarization ST trials after observation of two different expert demonstrations of skill<br>- 50 ST practice trials<br>*Day 1: Test phase:*<br>- 2 test blocks of 5 trials:<br>○ ST<br>○ DT (random letter generation) | ***Primary outcome:***<br>- Primary motor task: Task completion time (seconds; M±SD)<br>- Secondary task: Performance not assessed<br><br>***Secondary outcome:***<br>- Declarative knowledge: Number of explicit rules (M±SD) | **Motor task performance:**<br>*Implicit*<br>- ST = 211.3±45.1<br>- DT = 214.8±45.3<br>- DTC= 1.7%<br>*Explicit*<br>- ST = 202.2±37.8<br>- DT = 238.1±62.8<br>- DTC= 17.8%<br><br>**Secondary task performance: N/A**<br>**Declarative knowledge:**<br>*Implicit* = 2.8±1.8<br>*Explicit* = 5.3±3.4<br><br>**Implicit versus Explicit comparison:**<br>- Motor ST: *p*=.60<br>- Motor DT: *p*=.31<br>- Motor DTC: *p*=.12<br>- Secondary DT: N/A<br>- Secondary DTC: N/A<br>- Declarative knowledge: *p*=.03 |
| Masters et al. (2008b) [45] | **Number at baseline: 41 (6 drop-outs)**<br><br>**Inclusion/exclusion criteria:**<br>- Not specified<br><br>**Number of groups:** 2<br>- Errorless (n=17)<br>- Errorful (n=18) | Rugby passing task<br>- Rugby ball needed to be thrown underhand at an elevated target (125 cm) consisting of 3 | **Groups of interest:**<br>- Errorless ('Implicit'): Distance from target was progressively increased (1.0-3.0 m in 0.5 m steps)<br>- Errorful ('Explicit'): Distance from target was progressively reduced (6.0-4.0 m in 0.5 m steps)<br><br>**Procedure:** | ***Primary outcome:***<br>- Primary motor task: Accuracy (millimeters from target; M±SD)<br>- Secondary task: Performance not assessed<br><br>***Secondary outcome:*** | **Motor task performance:**<br>*Implicit*<br>- ST = 188.2±43.1<br>- DT = 184.0±37.5<br>- DTC= -2.2%<br>*Explicit*<br>- ST = 171.6±47.7<br>- DT = 211.2±39.0<br>- DTC= 23.1% |

| | | | | | |
|---|---|---|---|---|---|
| | **General descriptives:**<br>- Gender (m/f): ?<br>- Age (years): 20.5±1.2<br><br>**Specific group characteristics:** N/A<br><br>**Pre-test single task motor performance:** N/A | concentric squares (30, 100 & 150 cm) | *Day 1: Learning phase:*<br>- 100 ST trials<br>*Day 1: Test phase:*<br>- 4 test blocks of 10 trials, from 3.5 m distance:<br>o 2 ST<br>o 1 DT (random letter generation)<br>o 1 transfer block (10 ST trials after fatigued performance test on a treadmill) | - Declarative knowledge: No declarative knowledge assessment | **Secondary task performance: N/A**<br>**Declarative knowledge: N/A**<br><br>**Implicit versus Explicit comparison:**<br>- Motor ST: *p*=.29<br>- Motor DT: *p*=.04<br>- Motor DTC: *p*<.05<br>- Secondary DT: N/A<br>- Secondary DTC: N/A<br>- Declarative knowledge: N/A |
| Maxwell et al. (2001) [31]<br>***Experiment 1*** | **Number at baseline: 29**<br><br>**Inclusion/exclusion criteria:**<br>- No golfing experience<br><br>**Number of groups:** 3<br>- Errorless (n=11)<br>- Errorful (n=9)<br>- Control (n=9)<br><br>**General descriptives:**<br>- Gender (m/f): ?<br>- Age (years): 20.9±2.4<br><br>**Specific group characteristics:** N/A<br><br>**Pre-test single task motor performance:** N/A | Golf putting task | **Groups of interest:**<br>- Errorless ('Implicit'): Distance from target was progressively increased (0.25-2.0 m in 0.25 m steps)<br>- Errorful ('Explicit'): Distance from target was progressively reduced (2.0-0.25 m in 0.25 m steps)<br><br>**Procedure:**<br>*Day 1: Learning phase:*<br>- 8 blocks of 50 ST trials<br>*Day 1: Test phase:*<br>- 3 blocks of 50 trials, from 2 m distance:<br>o 1 ST<br>o 1 DT (tone counting task)<br>o 1 transfer block from novel distance of 3 m | ***Primary outcome:***<br>Primary motor task: Number of successful puts (M±SD)<br>- Secondary task: Accuracy (%; M±SD)<br><br>***Secondary outcome:***<br>- Declarative knowledge: Number of explicit rules (M±SD) | **Motor task performance:**<br>*Implicit*<br>- ST = 41.4±6.6<br>- DT = 41.6±6.7<br>- DTC= -0.6%<br>*Explicit*<br>- ST = 36.6±4.8<br>- DT = 32.8±5.9<br>- DTC= 10.3%<br><br>**Secondary task performance:**<br>*Implicit*<br>- ST = N/A<br>- DT = 93.3±8.8%<br>*Explicit*<br>- ST = N/A<br>- DT = 94.9±6.9%<br><br>**Declarative knowledge:**<br>*Implicit*= 4.3±1.6<br>*Explicit* = 3.1±1.5<br><br>**Implicit versus Explicit comparison:**<br>- Motor ST: *p*=.086<br>- Motor DT: *p*=.006<br>- Motor DTC: *p*=.047<br>- Secondary DT: *p*=.66<br>- Secondary DTC: N/A<br>- Declarative knowledge: *p*=.10 |

| | | | | | |
|---|---|---|---|---|---|
| Maxwell et al. (2001) [31]<br>***Experiment 2*** | **Number at baseline: 55**<br><br>**Inclusion/exclusion criteria:**<br>- No previous golfing experience<br><br>**Number of groups:** 4<br>- Errorless experimental (n=14)<br>- Errorless control (n=13)<br>- Errorful experimental (n=14)<br>- Errorful control (n=14)<br><br>**General descriptives:**<br>- Gender (m/f): ?<br>- Age (years): 21.0±2.8<br><br>**Specific group characteristics:** N/A<br><br>**Pre-test single task motor performance:** N/A | Golf putting task | **Groups of interest:**<br>- Errorless ('Implicit'): Distance from target was progressively increased (0.25-0.75 m in 0.25 m steps)<br>- Errorful ('Explicit'): Distance from target was progressively reduced (1.75-1.25 m in 0.25 m steps)<br><br>**Procedure:**<br>*Day 1: Learning phase:*<br>- 3 blocks of 50 ST trials<br>*Day 1: Test phase:*<br>- 1 block of 50 trials, from 1 m distance<br>o ST: errorless & errorful control groups<br>o DT (tone counting task): errorless & errorful experimental groups | ***Primary outcome:***<br>Primary motor task: Number of successful puts (M±SD)<br>- Secondary task: Accuracy (%; M±SD)<br><br>***Secondary outcome:***<br>- Declarative knowledge: Number of explicit rules + hypotheses (M±SD) | **Motor task performance:**<br>*Implicit*<br>- ST (errorless control) = 39.4<br>- DT (errorless exp) = 34.4<br>- DTC = N/A<br>*Explicit*<br>- ST (errorful control) = 35.5<br>- DT (errorful exp)= 30.6<br>- DTC = N/A<br><br>**Secondary task performance:**<br>*Implicit*<br>- ST (errorless control) = N/A<br>- DT (errorless exp)= 95.2±5.4<br>- DTC = N/A<br>*Explicit*<br>- ST (errorful control) = N/A<br>- DT (errorful exp)= 95.3±4.9<br>- DTC = N/A<br><br>**Declarative knowledge:**<br>*Implicit*:<br>- Errorless control = 3.2<br>- Errorless exp = 4.1<br>*Explicit*<br>- Errorful control = 5.3<br>- Errorful exp = 6.1<br><br>**Implicit versus Explicit comparison:**<br>- Motor ST: ?<br>- Motor DT: ?<br>- Motor DTC: N/A<br>- Secondary DT: *p*=.94<br>- Secondary DTC: N/A<br>- Declarative knowledge (combined errorless vs combined errorful groups): *p*>.05 |
| Maxwell et al. (2002) [46]<br>***Experiment 1*** | **Number at baseline: 20**<br><br>**Inclusion/exclusion criteria:**<br>- No experience with surfing, windsurfing or | Balancing task - Keeping a stabilometer (wobble board) horizontal | **Groups of interest:**<br>- External focus ('Implicit'):<br>o Augmented feedback of deviation of balance board (red dot)<br>o "Keep the board within the target | ***Primary outcome:***<br>- Primary motor task: Time on target (seconds) (M± SD)<br>- Secondary task: | **Motor task performance:**<br>*Implicit*<br>- ST = 85.84<br>- DT = 86.85<br>- DTC= -1.2% |

| | | | | | |
|---|---|---|---|---|---|
| | skate boarding (i.e., skills similar to the wobble board task)<br><br>**Number of groups:** 2<br>- External focus (n=10)<br>- Internal focus (n=10)<br><br>**General descriptives:**<br>- Gender (m/f): ?<br>- Age (years): 21.2±4.2<br><br>**Specific group characteristics:** N/A<br><br>**Pre-test single task motor performance:** N/A | | circle"<br>- Internal focus ('Explicit'):<br>o Augmented feedback of deviation of their feet (same red dot)<br>o "Keep your feet within the target circle"<br><br>**Procedure:**<br>*Day 1: Learning phase:*<br>- Ten 90-second ST trials<br>*Day 1: Test phase:*<br>- 2 blocks of three 90-second trials<br>o ST<br>o DT (tone counting task) | Accuracy (%; M±SD)<br><br>***Secondary outcome:***<br>- Declarative knowledge: Number of explicit (combined internal and external focus) rules (M±SD) | *Explicit*<br>- ST = 84.71<br>- DT = 86.31<br>- DTC= -1.9%<br><br>**Secondary task performance:**<br>*Implicit*<br>- ST = N/A<br>- DT = 94.64%<br>*Explicit*<br>- ST = N/A<br>- DT = 91.54%<br><br>**Declarative knowledge:**<br>*Implicit*: 1.7<br>*Explicit*: 2.0<br><br>**Implicit versus Explicit comparison:**<br>- Motor ST: *p*>.05<br>- Motor DT: *p*>.05<br>- Motor DTC: *p*>.05<br>- Secondary DT: *p*>.05<br>- Secondary DTC: N/A<br>- Declarative knowledge: *p*>.05 |
| Maxwell et al (2002) [46]<br>***Experiment 2*** | **Number at baseline: 20**<br><br>**Inclusion/exclusion criteria:**<br>- No previous golfing experience<br><br>**Number of groups:** 2<br>- External focus (n=10)<br>- Internal focus (n=10)<br><br>**General descriptives:**<br>- Gender (m/f): ?<br>- Age (years): 22.2±4.0<br><br>**Specific group characteristics:** N/A | Balancing task - Keeping a 2-axial stabilometer horizontal | **Groups of interest:**<br>- External focus ('Implicit'): "Keep the board horizontal"<br>- Internal focus ('Explicit'): "Keep your feet horizontal"<br><br>**Procedure:**<br>*Day 1: Learning phase:*<br>- Ten 90-second ST trials<br>*Day 1: Test phase:*<br>- 2 blocks of three 90-second trials<br>o ST<br>o DT (tone counting task) | ***Primary outcome:***<br>- Primary motor task:<br>o Pitch (RMSE backward-forward deviations; M±SD)<br>o Roll (RMSE medio-lateral deviations; M±SD)<br>- Secondary task: Accuracy (%; M± SD)<br><br>***Secondary outcome:***<br>- Declarative knowledge: Number of explicit (combined internal and external focus) rules (M± | **Motor task performance:**<br>*Implicit*<br>- ST ('Pitch') = 18.0<br>- DT ('Pitch') = 18.2<br>- DTC ('Pitch') = 1.1%<br>- ST ('Roll') =18.4<br>- DT ('Roll') =18.2<br>- DTC('Roll') = -1.1%<br>*Explicit*<br>- ST ('Pitch') = 20.1<br>- DT ('Pitch') = 21.1<br>- DTC ('Pitch') = 5.0%<br>- ST ('Roll') = 20.3<br>- DT ('Roll') = 20.2<br>- DTC('Roll') = -0.5% |

| | | | | | |
|---|---|---|---|---|---|
| | **Pre-test single task motor performance:** N/A | | | SD) | **Secondary task performance:**<br>*Implicit*<br>- ST = N/A<br>- DT = 95.83%<br>- DTC = N/A<br>*Explicit*<br>- ST = N/A<br>- DT = 94.14%<br>- DTC = N/A<br><br>**Declarative knowledge:**<br>*Implicit*: 2.1<br>*Explicit*: 1.8<br><br>**Implicit versus Explicit comparison:**<br>- Motor ST: $p$>.05<br>- Motor DT: $p$>.05<br>- Motor DTC: $p$>.05<br>- Secondary DT: $p$>.05<br>- Secondary DTC: N/A<br>- Declarative knowledge: $p$>.05 |
| Orrell et al. (2006a) [48] | **Number at baseline: 24 (2 drop-outs in stroke groups)**<br><br>**Inclusion/exclusion criteria:**<br>- No neurological impairment<br><br>**Number of groups:** 4<br>- Errorless–stroke (n=5)<br>- Errorless–control (n=6)<br>- Discovery-stroke (n=5)<br>- Discovery–control (n=6)<br><br>**Specific group characteristics:**<br>- Errorless-stroke<br>  o Gender (m/f): 4/1<br>  o Age (years): 49.2±15.7 | Balancing task - Keeping a 1-axial stabilometer horizontal | **Groups of interest:**<br>- Errorless-control ('Implicit'): Progressively reduced braking resistance (2.5kg-0kg in 0.5kg steps)<br>- Discovery-control ('Explicit'): Instruction to discover rules of how to perform the balancing task<br><br>**Procedure:**<br>*Day 1: Acquisition phase:*<br>- Twenty-four 60-second ST trials<br>*Day 1:Test phase 1: (no braking resistance)*<br>- 4 ST-retention trials<br>- 2 ST-DT "Recall" trials<br>  o $1^{st}$ 30 seconds: ST<br>  o $2^{nd}$ 30 seconds: DT (recall random 6-digit sequences)<br>- 2 ST-DT "Kettle" trials | ***Primary outcome:***<br>- Primary motor task: RMSE of deviation from horizontal (M± SD)<br>- Secondary tasks: Performance not assessed<br><br>***Secondary outcome:***<br>- Declarative knowledge: Number of explicit rules reported (M± SD) | **Motor task performance:**<br>*Implicit*<br>- ST ($1^{st}$ 30 s of ST-DT trial)<br>  o Recall = 6.09±0.23<br>  o Kettle = 5.79±0.25<br>- DT ($2^{nd}$ 30 s of ST-DT trial)<br>  o Recall = 6.25±0.36<br>  o Kettle = 6.08±0.27<br>- DTC ('Recall') = 2.6%<br>- DTC ('Kettle') = 5.0%<br>*Explicit*<br>- ST ($1^{st}$ 30 s of ST-DT trial)<br>  o Recall = 6.35±0.43<br>  o Kettle = 5.96±0.42<br>- DT ($2^{nd}$ 30 s of ST-DT trial)<br>  o Recall = 6.37±0.38<br>  o Kettle = 5.81±0.33<br>- DTC ('Recall') = 0.3% |

| | | | | | |
|---|---|---|---|---|---|
| | ○ MMSE: 26.8±0.8<br>○ BBS: 38.4±5.8<br>- Errorless-control:<br>○ Gender (m/f): 3/3<br>○ Age (years): 67.2±8.7<br>○ MMSE: 29.2±0.7<br>○ BBS: 52.3±1.4<br>- Discovery-stroke<br>○ Gender (m/f): 5/0<br>○ Age (years): 54.6±12.2<br>○ MMSE: 25.8±1.3<br>○ BBS: 38.0±9.0<br>- Discovery-control<br>○ Gender (m/f): 3/3<br>○ Age (years): 63.2±5.3<br>○ MMSE: 29.3±0.8<br>○ BBS: 53.5±0.8<br><br>**Pre-test single task motor performance:** N/A | | ○ 1st 30 seconds: ST<br>○ 2nd 30 seconds: DT (reach out and pick up a 1-kg kettle with 1 hand)<br>*Day 7: Test phase 2:(no braking resistance)*<br>- 2 ST trials | | - DTC ('Kettle') = -2.5%<br><br>**Secondary task performance: N/A**<br><br>**Declarative knowledge:**<br>*Implicit* = 1.8±0.8<br>*Explicit* = 2.7±1.0<br><br>**Implicit versus Explicit comparison:**<br>- Motor ST:<br>○ Recall: *p*=.27<br>○ Kettle: *p*=.46<br>- Motor DT:<br>○ Recall: *p*=.59<br>○ Kettle: *p*=.15<br>- Motor DTC<br>○ Recall: *p*=>.05<br>○ Kettle: *p*=>.05<br>- Secondary DT: N/A<br>- Secondary DTC: N/A<br>- Declarative knowledge: *p* = 0.12 |
| Orrell et al. (2006b) [47] | **Number at baseline: 42 (6 drop-outs)**<br><br>**Inclusion/exclusion criteria:**<br>- No previous experience of surfing, snowboarding or other similar balancing tasks<br><br>**Number of groups:** 3<br>- Analogy (n=11)<br>- Errorless (n=13)<br>- Discovery (n=12)<br><br>**General descriptives:**<br>- Gender (m/f): 17/19<br>- Age (years): 20.3±1.2<br><br>**Specific group characteristics:** | Balancing task - Keeping a 1-axial stabilometer horizontal | **Groups of interest:**<br>- Analogy ('Implicit'): "Pretend to be soldiers standing on guard outside Buckingham Palace"<br>- Errorless ('Implicit'): Amount of available displacement from horizontal axis was gradually increased every second trial<br>- Discovery learning ('Explicit'): Instructions to discover rules of how to perform the balancing task<br><br>**Procedure:**<br>*Day 1: Acquisition phase:*<br>- Sixteen 60-second ST trials<br>*Day 1: Test phase 1:*<br>- 4 ST trials<br>- 2 ST-DT "Recall" trials<br>○ 1st 30 seconds: ST<br>○ 2nd 30 seconds: DT (recall random | ***Primary outcome:***<br>- Primary motor task: RMSE of deviation from horizontal (M± SD)<br>- Secondary task:<br>○ Recall & Kettle: Performance not assessed<br>○ Tone counting: Accuracy (%; M± SD)<br><br>***Secondary outcome:***<br>- Declarative knowledge: Number of explicit rules (M± SD) | **Motor task performance:**<br>***Test phase 1 (Immediate)***<br>*Implicit*<br>- ST (1st 30 s of ST-DT trial)<br>○ Analogy:<br>Recall = 3.51±0.20<br>Kettle = 3.25±0.21<br>○ Errorless:<br>Recall = 2.91±0.46<br>Kettle = 2.81±0.48<br>- DT (2nd 30 s of ST-DT trial)<br>○ Analogy:<br>Recall = 3.26±0.26<br>Kettle = 3.55±0.20<br>○ Errorless:<br>Recall = 2.77±0.49<br>Kettle = 2.94±0.50<br>- DTC Analogy ('Recall') = -7.1%<br>- DTC Errorless ('Recall') = -4.8%<br>- DTC Analogy ('Kettle') = 9.2% |

| | | | | | |
|---|---|---|---|---|---|
| | - Analogy:<br>  o Gender (m/f): 5/6<br>- Errorless:<br>  o Gender (m/f): 6/7<br>- Discovery:<br>  o Gender (m/f): 6/6<br><br>**Pre-test single task motor performance:** N/A | | 7-digit sequence)<br>- 2 ST-DT "Kettle" trials<br>  o 1st 30 seconds: ST<br>  o 2nd 30 seconds: DT (reach out and pick up a 1-kg kettle with 1 hand)<br>*Day 15: Test phase 2:*<br>- 2 ST trials<br>- 2 ST-DT trials "Count+Kettle"<br>  o 1st 30 seconds: tone counting<br>  o 2nd 30 seconds: tone counting + kettle-lifting task | | - DTC Errorless ('Kettle') = 4.6%<br><br>*Explicit*<br>- ST (1st 30 s of ST-DT trial)<br>  o Recall = 2.84±0.36<br>  o Kettle = 2.74±0.41<br>- DT (2nd 30 s of ST-DT trial)<br>  o Recall = 2.55±0.31<br>  o Kettle = 2.85 ±0.36<br>- DTC ('Recall') = -10.2%<br>- DTC ('Kettle') = 4.0%<br><br>***Test phase 2 (Delayed)***<br>*Implicit*<br>- ST<br>  o Analogy = 3.44±0.29<br>  o Errorless = 2.96±0.34<br>- DT<br>  o Analogy:<br>    Count = 3.59±0.28<br>    Count+Kettle = 3.46±0.28<br>  o Errorless:<br>    Count = 2.59±0.43<br>    Count+Kettle = 2.69±0.43<br>- DTC Analogy ('Count') = 4.4%<br>- DTC Errorless ('Count')= -12.5%<br>- DTC Analogy ('Count+Kettle') = 0.6%<br>- DTC Errorless ('Count+Kettle') = -9.1%<br><br>*Explicit*<br>- ST = 2.95±0.47<br>- DT<br>    Count = 2.92±0.46<br>    Count+Kettle = 2.44±0.45<br>- DTC ('Count') = -1.0%<br>- DTC ('Count+Kettle') = -17.3%<br><br>**Secondary task performance: N/A**<br><br>**Declarative knowledge:** |

| | | | | | |
|---|---|---|---|---|---|
| | | | | | *Implicit*<br>○ Analogy = 1.6±1.1<br>○ Errorless = 2.1±1.0<br>*Explicit* = 2.8±0.8<br><br>**Implicit versus Explicit comparison:**<br>***Test phase 1 (Immediate):***<br>Analogy vs Explicit<br>- Motor ST:<br>○ Recall: *p*<.0001<br>○ Kettle: *p*=.0001<br>- Motor DT:<br>○ Recall: *p*<.0001<br>○ Kettle: *p*<.0001<br>- Motor DTCs<br>○ Recall: *p*=.84<br>○ Kettle: *p*=.81<br>- Secondary DT: N/A<br>- Secondary DTC: N/A<br>Errorless vs Explicit<br>- Motor ST:<br>○ Recall: *p*=.68<br>○ Kettle: *p*=.70<br>- Motor DT:<br>○ Recall: *p*=.20<br>○ Kettle: *p*=.61<br>- Motor DTCs<br>○ Recall: *p*=.84<br>○ Kettle: *p*=.81<br>- Secondary DT: N/A<br>- Secondary DTC: N/A<br>***Test phase 2 (Delayed):***<br>Analogy vs Explicit<br>- Motor ST: *p*=0.005<br>- Motor DT:<br>○ Counting: *p*<.001<br>○ Counting+Kettle: *p*<.0001<br>- Motor DTCs: ?<br>- Secondary DT (Counting): *p*>.11<br>- Secondary DTC: N/A<br>Errorless vs Explicit<br>- Motor ST: *p*=0.95 |

| | | | | | |
|---|---|---|---|---|---|
| | | | | | - Motor DT:<br>○ Counting: *p*=.08<br>○ Counting+Kettle: *p*=.17<br>- Motor DTCs: ?<br>- Secondary DT (Counting): *p*>.11<br>- Secondary DTC: N/A<br>***Test Phase 1+2:***<br>- Declarative knowledge:<br>○ Analogy vs Explicit: *p* = .008<br>○ Errorless vs Explicit: *p* = .048 |
| Poolton et al. (2005) [49] | **Number at baseline: 35**<br><br>**Inclusion/exclusion criteria:**<br>- No previous golf putting experience<br><br>**Number of groups:** 2<br>- Implicit-Explicit (n=17)<br>- Explicit (n=18)<br><br>**General descriptives:**<br>- Gender (m/f): 11/24<br>- Age (years): 21.1±1.5<br><br>**Specific group characteristics:** N/A<br><br>**Pre-test single task motor performance:** N/A | Golf putting task | **Groups of interest:**<br>- Implicit-Explicit (‘Implicit’): Distance from target was progressively increased (0.25-2.0 m in 0.25 m steps); After fourth block of trials, a set of 6 putting technique instructions was provided<br>- Explicit: Distance from target was progressively increased (0.25-2.0 m in 0.25 m steps) Different from the implicit group, the 6 technical putting instructions were provided from start of learning<br><br>**Procedure:**<br>*Day 1: Learning phase:*<br>- 8 blocks of 50 trials (400 trials):<br>*Day 1: Test phase:*<br>- 3 test blocks of 50 trials, all at 2 m<br>○ 2 ST<br>○ 1 DT (tone counting) | ***Primary outcome:***<br>- Primary motor task: Number of successful putts (M± SD)<br>- Secondary task: Tone counting accuracy (%; M± SD)<br><br>***Secondary outcome:***<br>Declarative knowledge:<br>- Number of explicit rules (M± SD) | **Motor task performance:**<br>*Implicit*<br>- ST = 28.23<br>- DT = 31.11<br>- DTC= -10.2%<br>*Explicit*<br>- ST = 29.98<br>- DT = 26.19<br>- DTC=12.6%<br><br>**Secondary task performance:**<br>*Implicit*<br>- Single task = N/A<br>- Dual-task = 94.1%<br>- DTC = N/A<br>*Explicit*<br>- Single task = N/A<br>- Dual-task = 92.8%<br>- DTC = N/A<br><br>**Declarative knowledge:**<br>*Implicit* = 4.1<br>*Explicit* = 5.2<br><br>**Implicit versus Explicit comparison:**<br>- Motor ST: ?<br>- Motor DT: ?<br>- Motor DTC: *p*<.01<br>- Secondary DT: *p*=.68<br>- Secondary DTC: N/A<br>- Declarative knowledge: *p*>.05 |

| | | | | | |
|---|---|---|---|---|---|
| Poolton et al. (2006) [50]<br>***Experiment 1*** | **Number at baseline: 30**<br><br>**Inclusion/exclusion criteria:**<br>- No experience with golf putting<br><br>**Number of groups:** 2<br>- Internal focus (n=15)<br>- External focus (n=15)<br><br>**General descriptives:**<br>- Gender (m/f): 7/23<br>- Age (years): 24.1±5.9<br><br>**Specific group characteristics:** N/A<br><br>**Pre-test single task motor performance:** N/A | Golf putting task | **Groups of interest:**<br>- Internal focus ('Explicit'): "Direct attention to the swing of your hands"<br>- External focus ('Implicit'): "Focus on swing of the putter head"<br><br>**Procedure:**<br>*Day 1: Learning phase:*<br>- 10 blocks of 30 ST trials<br>*Day 1: Test phase:*<br>- 3 blocks of 30 trials<br>  o 2 ST<br>  o 1 DT (tone counting) | ***Primary outcome:***<br>- Primary motor task: Number of successful putts (M± SD)<br>- Secondary task: Tone counting accuracy (%; M± SD)<br><br>***Secondary outcome:***<br>- Declarative knowledge: Number of explicit (combined internal/external) rules (M± SD) | **Motor task performance:**<br>*Implicit*<br>- ST = 9.08<br>- DT = 9.81<br>- DTC= -8.0%<br>*Explicit*<br>- ST = 9.44<br>- DT = 7.30<br>- DTC= 22.7%<br><br>**Secondary task performance:**<br>*Implicit*<br>- ST = N/A<br>- DT = 91.2%<br>- DTC = N/A<br>*Explicit*<br>- ST = N/A<br>- DT = 93.3%<br>- DTC = N/A<br><br>**Declarative knowledge:**<br>*Implicit:* 4.0<br>*Explicit*: 5.3<br><br>**Implicit versus Explicit comparison:**<br>- Motor ST: $p$=0.54<br>- Motor DT: $p$<.05<br>- Motor DTC: $p$<.05<br>- Secondary DT: $p$=.42<br>- Secondary DTC: N/A<br>- Declarative knowledge: $p$<.05 |
| Poolton et al. (2006) [50]<br>***Experiment 2*** | **Number at baseline: 39**<br><br>**Inclusion/exclusion criteria:**<br>- No experience with golf putting<br><br>**General descriptives:**<br>- Gender (m/f): 15/24<br>- Age (years): 20.4±3.8 | Golf putting task. | **Groups of interest:**<br>- Internal focus ('Explicit'): Received 6 instructions regarding desired movement of their hands<br>- External focus ('Implicit'): Received 6 instructions regarding desired movement of the club<br><br>**Procedure:**<br>Identical to experiment 1 | ***Primary outcome:***<br>- Primary motor task: Number of successful putts (M± SD)<br>- Secondary task: Tone counting accuracy (%; M± SD)<br><br>***Secondary outcome:***<br>- Declarative knowledge: | **Motor task performance:**<br>*Implicit*<br>- ST = 41.08<br>- DT = 27.77<br>- DTC= 32.4%<br>*Explicit*<br>- ST = 41.83<br>- DT = 35.11<br>- DTC= 16.1% |

| | | | | | |
|---|---|---|---|---|---|
| | **Number of groups:** 2;<br>- Internal focus (n=19)<br>- External focus (n=20)<br><br>**Specific group characteristics:** N/A<br><br>**Pre-test single task motor performance:** N/A | | | Number of explicit (combined internal, external, and neutral) rules (M± SD) | **Secondary task performance:**<br>*Implicit*<br>- ST = N/A<br>- DT = 89.4<br>- DTC = N/A<br>*Explicit*<br>- ST = N/A<br>- DT = 88.4<br>- DTC = N/A<br><br>**Declarative knowledge:**<br>*Implicit*: 5.5<br>*Explicit:* 4.8<br><br>**Implicit versus Explicit comparison:**<br>- Motor ST: $p$>.56<br>- Motor DT: $p$>.56<br>- Motor DTC: $p$=.42<br>- Secondary DT: $p$=.81<br>- Secondary DTC: N/A<br>- Declarative knowledge: $p$=.19 |
| Poolton et al. (2007a) [51] | **Number at baseline: 56 (1 person excluded in errorful group)**<br><br>**Inclusion/exclusion criteria:**<br>- No previous experience with rugby throwing task<br><br>**Number of groups:** 2<br>- Errorless (n=23)<br>- Errorful (n=22)<br>- Control (n=10)<br><br>**Specific group characteristics:**<br>- Errorless & Errorful combined:<br>o Gender (m/f): 23/22<br>o Age (years): 23.0±5.0<br>- Control<br>o Gender (m/f) = 4/6 | Rugby throwing task<br>- Rugby ball needed to be thrown underhand at an elevated target (125 cm) consisting of 3 concentric squares (30, 100, & 150 cm). | **Groups of interest:**<br>- Errorless ('Implicit'): Distance from target was progressively increased (1.0-3.0 m in 0.5 m steps)<br>- Errorful ('Explicit'): Distance from target was progressively reduced (6.0-4.0 m in 0.5 m steps)<br><br>**Procedure:**<br>*Day 1: Learning phase:*<br>- 10 blocks of 10 trials<br>*Day 1: Test phase:*<br>- 4 test blocks of 10 trials, at 3.5 m:<br>o 2 ST<br>o 1 DT (random letter generation)<br>o 2 Transfer blocks (ST after fatigued-performance test)<br>- *One year later: Test phase 2:*<br>o 1 ST block (10 trials at 3.5 m) | ***Primary outcome:***<br>- Primary motor task: Distance from target in mm (M± SD)<br>- Secondary task: Performance not assessed<br><br>***Secondary outcome:***<br>- Declarative knowledge: Number of explicit rules (M± SD) | **Motor task performance:**<br>*Implicit*<br>- ST = 213.3<br>- DT = 200.5<br>- DTC= -6.0%<br>*Explicit*<br>- ST = 189.7<br>- DT = 240.4<br>- DTC= 26.7%<br><br>**Secondary task performance: N/A**<br><br>**Declarative knowledge:**<br>*Implicit* = 2.4±1.8<br>*Explicit* = 4.5±2.9<br><br>**Implicit versus Explicit comparison:**<br>- Motor ST: $p$>.05<br>- Motor DT: $p$=0.06<br>- Motor DTC: $p$<.01 |

| | | | | | |
|---|---|---|---|---|---|
| | o Age = 29.0±5.3<br><br>**Pre-test single task motor performance:** N/A | | | | - Secondary DT: N/A<br>- Secondary DTC: N/A<br>- Declarative knowledge: *p*<.005 |
| Poolton et al. (2007b) [52] | **Number at baseline: 28**<br><br>**Inclusion/exclusion criteria:**<br>- Not having received formal table tennis coaching or played table tennis more than once a month<br>- Cantonese as first language<br>- Right hand dominant<br><br>**Number of groups:** 2<br>- Analogy (n=14)<br>- Explicit (n=14)<br><br>**General descriptives:**<br>- Gender (m/f): ?<br>- Age (years): ?<br><br>**Specific group characteristics:** N/A<br><br>**Pre-test single task motor performance:** N/A | Table tennis task<br>- Hit table tennis ball onto target area with topspin using forehand stroke | **Groups of interest:**<br>- Analogy ('Implicit'): "Move the bat as though it is traveling up the side of a mountain."<br>- Explicit: 6 technical table tennis forehand instructions<br><br>**Procedure:**<br>*Day 1: Learning phase:*<br>- 300 ST trials<br>*Day 1: Test phase:*<br>- 3 test blocks<br>o 2 ST<br>o 1 DT (counting backwards) | ***Primary outcome:***<br>- Primary motor task: Number of points scored (M± SD)<br>- Secondary task: Performance not assessed<br><br>***Secondary outcome:***<br>- Declarative knowledge: Number of explicit rules (M± SD) | **Motor task performance:**<br>*Implicit*<br>- ST = 18.53<br>- DT = 17.67<br>- DTC= 4.6%<br>*Explicit*<br>- ST = 19.27<br>- DT = 13.76<br>- DTC= 28.6%<br><br>**Secondary task performance: N/A**<br><br>**Declarative knowledge:**<br>*Implicit* = 3.3<br>*Explicit* = 5.8<br><br>**Implicit versus Explicit comparison:**<br>- Motor ST: ?<br>- Motor DT: ?<br>- Motor DTC: *p*<.05<br>- Secondary DT: N/A<br>- Secondary DTC: N/A<br>- Declarative knowledge: *p*<.001 |
| Sanli et al (2014) [53]<br>***Experiment 1*** | **Number at baseline: 19**<br><br>**Inclusion/exclusion criteria:**<br>- Not specified<br><br>**Number of groups:** 2<br>- Errorless (n=10)<br>- Errorful (n=9)<br><br>**General descriptives:**<br>- Gender (m/f): 10/9<br>- Age (years) 25.6±3.2<br><br>**Specific group** | Aiming task<br>- Subjects needed to propel small disc over a smooth table top, with the aim to stop it in a specified target circle | **Groups of interest:**<br>- Errorless ('Implicit'): Progressively reduced target size (i.e., 31-6.5 cm diameter, in 3.5 cm steps)<br>- Errorful ('Explicit'): Progressively increased target size (i.e., 6.5-31 cm diameter, in 3.5 cm steps)<br><br>**Procedure:**<br>*Day 1: Acquisition phase:*<br>- 200 trials (25 to each of eight target sizes)<br>*Day 1: Test phase 1:* | ***Primary outcome:***<br>- Primary motor task: Proportion of errors (i.e., number of times the disc did not stop completely within the target area) (M± SD)<br>- Secondary task: Tone counting accuracy (%; M± SD)<br><br>***Secondary outcome:***<br>- Declarative knowledge: Not assessed | **Motor task performance:**<br>***Test phase 1 (Immediate)***<br>*Implicit*<br>- ST = 0.81<br>- DT = 0.76<br>- DTC= -6.2%<br>*Explicit*<br>- ST = 0.82<br>- DT = 0.75<br>- DTC= -8.5%<br>***Test phase 2 (Delayed)***<br>*Implicit*<br>- ST = 0.83<br>- DT = 0.81 |

| | | | | | |
|---|---|---|---|---|---|
| | **characteristics:** N/A<br><br>**Pre-test single task motor performance:** N/A | | - 2 test blocks of 25 trials, with 6.5 cm target size<br>o 1 ST<br>o 1 DT (tone counting)<br>- 1 Transfer test block of 25 trials, with a 4.5 cm target size that had not been practiced<br>*Day 2: Test phase 2:*<br>Identical to test phase 1. | | - DTC= -2.4%<br>*Explicit*<br>- ST = 0.81<br>- DT = 0.81<br>- DTC= 0%<br><br>**Secondary task performance:** ?<br>**Declarative knowledge:** N/A<br><br>**Implicit versus Explicit comparison (Test phase 1 & 2 combined):**<br>- Motor ST: *p*>.05<br>- Motor DT: *p*>.05<br>- Motor DTC: *p*>.05<br>- Secondary DT: *p*>.05<br>- Secondary DTC: N/A<br>- Declarative knowledge: N/A |
| Sanli et al (2014) [53]<br>***Experiment 2*** | **Number at baseline: 20**<br><br>**Inclusion/exclusion criteria:**<br>- Not specified<br><br>**Number of groups:** 2<br>- Errorless (n=10)<br>- Errorful (n=10)<br><br>**General descriptives:**<br>- Gender (m/f) = 8/12<br>- Age (years) 21.2±2.9<br><br>**Specific group characteristics:** N/A<br><br>**Pre-test single task motor performance:** N/A | Aiming task<br>- Subjects needed to propel small disc over a smooth table top, with the aim to stop it in a specified target circle (6.5 cm diameter) | **Groups of interest:**<br>- Errorless ('Implicit'): Progressively increased distance from target (i.e., 3.5-7.5-11.5-15.5-18.5-22.5-26.5-30.5 cm distance)<br>- Errorful ('Explicit'): Progressively reduced distance from target (i.e., 30.5-26.5-22.5-18.5-15.5-11.5-7.5-3.5 cm distance)<br><br>**Procedure:**<br>Identical to Sanli 2014 – Experiment 1 | ***Primary outcome:***<br>- Primary motor task: Proportion of errors (i.e., number of times the disc did not stop completely within the target area) (M± SD)<br>- Secondary task: Tone counting accuracy (%; M± SD)<br><br>***Secondary outcome:***<br>- Declarative knowledge: Not assessed | **Motor task performance:**<br>***Test phase 1 (Immediate)***<br>*Implicit*<br>- ST = 0.89<br>- DT = 0.84<br>- DTC = -5.6%<br>*Explicit*<br>- ST = 0.88<br>- DT = 0.82<br>- DTC = -6.8%<br>***Test phase 2 (Delayed)***<br>*Implicit*<br>- ST = 0.95<br>- DT = 0.89<br>- DTC = -6.3%<br>*Explicit*<br>- ST = 0.87<br>- DT = 0.84<br>- DTC = -3.4%<br><br>**Secondary task performance:** ?<br>**Declarative knowledge:** N/A<br><br>**Implicit versus Explicit comparison (Test phase 1 & 2 combined):**<br>- Motor ST: *p*>.05 |

| | | | | | |
|---|---|---|---|---|---|
| | | | | | - Motor DT: *p*>.05<br>- Motor DTC: *p*>.05<br>- Secondary DT: *p*>.05<br>- Secondary DTC: N/A<br>- Declarative knowledge: N/A |
| Schücker et al. (2010) [54] | **Number at baseline:** 51 (5 drop outs)<br><br>**Inclusion/exclusion criteria:**<br>- Inexperienced in golf playing and had no official permission to play golf in Germany<br><br>**Number of groups:** 2<br>- Analogy (n=25)<br>- Technical learning (n=21)<br><br>**General descriptives:**<br>- Gender (m/f): 33/18<br>- Age (years): 32.7±12.3<br>- Handedness (r/l): 49/2<br><br>**Specific group characteristics:** N/A<br><br>**Pre-test single task motor performance:**<br>- Analogy<br>  o Carry (m)=70.4±25.3<br>  o Off-line(°)=12.9±5.5<br>- Technical learning<br>  o Carry (m)=80.2±26.6<br>  o Off-line(°)=12.4±3.6<br>***Between group pre-test comparison***<br>- 'Carry': *p*=.21<br>- 'Off-line': *p*=.73 | Full swing golf stroke | **Groups of interest:**<br>- Analogy ('Implicit'):<br>9 analogies on different aspects of position & 21 analogies on different aspects of swing; (e.g., "Imagine you have an open tube of toothpaste between your hands and the contents must not be pushed out" (grip))<br>- Technical ('Explicit'):<br>9 technical instructions on position & 21 technical instructions on golf swing<br><br>**Procedure:**<br>*Week 1-6 Learning phase:*<br>- 1 golf lesson/week from golf-professional+1 hour free practice<br>*Test Phases:*<br>- Pre (after 1st training):10 ST trials<br>- Post-ST (after 5th training): 10 ST trials<br>- Post-DT (after 6st training):<br>  o Tone judgment task (judging in which phase of swing tone was played)<br>  o 12 low-pressure DT trials<br>  o 12 high-pressure DT trials | ***Primary outcome:***<br>- Primary motor task:<br>  o "Carry" distance (m; M±SD)<br>  o "Off-line" flight deviance in degrees (M±SD)<br>- Secondary task:<br>Tone judgement accuracy (%; M± SD)<br><br>***Secondary outcome:***<br>- Declarative knowledge: Not assessed | **Motor task performance:**<br>*Implicit*<br>- ST<br>  o Carry (m) = 81.7±27.4<br>  o Off-line(°) = 12.2±5.0<br>- DT (low pressure)<br>  o Carry (m)=80.8±23.3<br>  o Off-line(°)=16.1±7.5<br>- DTC ('Carry') = 1.1%<br>- DTC ('Off-line') = 32.0%<br>*Explicit*<br>- ST<br>  o Carry (m) = 90.2±32.1<br>  o Off-line(°) = 12.9±5.7<br>- DT (low pressure)<br>  o Carry (m)=89.7±27.3<br>  o Off-line(°)=14.5±6.1<br>- DTC ('Carry') = 0.6%<br>- DTC ('Off-line') = 12.4%<br><br>**Secondary task performance:**<br>*Implicit*<br>- ST = N/A<br>- DT (low pressure) = 27.0±13.9<br>- DT = N/A<br>*Explicit*<br>- ST = N/A<br>- DT (low pressure) = 27.6±11.7<br>- DTC = N/A<br>-<br>**Declarative knowledge:** N/A<br><br>**Implicit versus Explicit comparison:**<br>- Motor ST<br>  o Carry: *p*=.34 |

| | | | | | |
|---|---|---|---|---|---|
| | | | | | ○ Off-line: *p*=.66<br>- Motor DT<br>○ Carry: *p*=.24<br>○ Off-line: *p*=.44<br>- Motor DTC: *p*= ?<br>- Secondary DT: *p*=.88<br>- Secondary DTC: N/A<br>- Declarative knowledge: N/A |
| Schücker et al. (2013) [55] | **Number at baseline: 41**<br><br>**Inclusion/exclusion criteria:**<br>- No previous golf experience<br>- Never having received formal golf putting instructions<br><br>**Number of groups:** 2<br>- Analogy (n=20)<br>- Technical learning (n=21)<br><br>**General descriptives:**<br>- Gender (m/f): 23/18<br>- Age (years): 21.4±3.0<br>- Handedness (r/l): 35/6<br><br>**Specific group characteristics:**<br>- Analogy:<br>○ Gender (m/f): 11/9<br>○ Handedness (r/l): 17/3<br>- Technical<br>○ Gender (m/f): 12/9<br>○ Handedness (r/l): 18/3<br><br>**Pre-test single task motor performance:** N/A | Golf putting task | **Groups of interest:**<br>- Analogy ('Implicit'): "Perform the putt like a pendulum (with visual instruction of a weight swinging on a cord)"<br>- Technical ('Explicit'): A set of 6 technical putting instructions<br><br>**Procedure:**<br>*Day 1: Learning phase:*<br>- 6 blocks of 50 ST trials (300 trials total)<br>*Day 1: Test phase*<br>- 4 blocks of 20 trials, all with DT (judging pitch of tone or movement phase when tone was played)<br>○ 1 familiarization DT block<br>○ 2 low pressure DT<br>○ 1 high pressure DT | ***Primary outcome:***<br>- Primary motor task: Mean distance from target (cm; M± SD)<br>- Secondary task:<br>○ Tone pitch judgment accuracy (%; M± SD)<br>○ Movement phase judgment accuracy (%; M± SD)<br><br>***Secondary outcome:***<br>- Declarative knowledge: Number of explicit rules (M± SD) | **Motor task performance:**<br>*Implicit*<br>- ST = N/A<br>- DT = 28.2±6.8<br>- DTC = N/A<br>*Explicit*<br>- ST = N/A<br>- DT = 27.9±7.3<br>- DTC =N/A<br><br>**Secondary task performance:**<br>*Implicit*<br>- ST = N/A<br>- DT<br>○ Pitch: 93.3±11.2<br>○ Movement phase: 0.83±0.38<br>- DTC = N/A<br>*Explicit*<br>- ST = N/A<br>- DT<br>○ Pitch: 92.8±12.1<br>○ Movement phase: 0.70±0.37<br>- DTC = N/A<br><br>**Declarative knowledge:**<br>*Implicit* = 2.0±1.0<br>*Explicit* = 3.4±1.2<br><br>**Implicit versus Explicit comparison:**<br>- Motor ST: N/A<br>- Motor DT: *p*=.89<br>- Motor DTC: N/A<br>- Secondary DT |

| | | | | | |
|---|---|---|---|---|---|
| | | | | | ○ Pitch: *p*=.89<br>○ Movement phase: *p*=.27<br>- Secondary DTC = N/A<br>- Declarative knowledge: *p*<.001 |
| Singer et al. (1993) [56] | **Number at baseline: 72**<br><br>**Inclusion/exclusion criteria:**<br>- Not specified<br><br>**Number of groups:** 4<br>- Awareness group (n=18)<br>- Non-awareness (n=18)<br>- 5-Step Approach (n=18)<br>- Control (n=18)<br><br>**General descriptives:**<br>- Gender (m/f): 36/36<br>- Age (years): 20.1±?<br><br>**Specific group characteristics:** N/A<br><br>**Pre-test single task motor performance:** N/A | Non-dominant overhand throwing task<br>- Subjects threw paddle balls via the ground at acircular target (diameter = 65.45 cm), from a 3.66 m distance | **Groups of interest:**<br>- Non-awareness ('Implicit'): Focus on one situational cue (e.g., center of the target) and to ignore movement information<br>- Awareness ('Explicit'): Instructions to be aware of the way that they threw the ball<br><br>**Procedure:**<br>*Days 1&2: Learning phase:*<br>- 5 blocks of 25 ST trials per day (250 in total)<br>*Day 2: Test phase*<br>- 50 DT trials (Subjects learned a 5-digit sequence before throwing & needed to call out one of these digits when prompted during throwing) | ***Primary outcome:***<br>- Primary motor task: Radial error (distance from target in degrees) (M± SD)<br>- Secondary task: Accuracy on digit recall task (%; M± SD)<br><br>***Secondary outcome:***<br>- Declarative knowledge: Not assessed | **Motor task performance:**<br>*Implicit*<br>- ST = N/A<br>- DT = ?<br>- DTC = N/A<br>*Explicit*<br>- ST = N/A<br>- DT = ?<br>- DTC = N/A<br><br>**Secondary task performance: ?**<br>**Declarative knowledge:** N/A<br><br>**Implicit versus Explicit comparison:**<br>- Motor ST: N/A<br>- Motor DT: *p*<.05<br>- Motor DTC: N/A<br>- Secondary DT: *p*>.05<br>- Secondary DTC: N/A<br>- Declarative knowledge: N/A |
| Totsika et al. (2003) [57] | **Number at baseline: 22**<br><br>**Inclusion/exclusion criteria:**<br>- No experience with Pedalo task<br><br>**Number of groups:** 2<br>- Internal focus (n=11)<br>- External focus (n=11)<br><br>**General descriptives:**<br>- Gender(m/f): 10/12<br>- Age (years): 23.4±?<br><br>**Specific group characteristics:**<br>- Internal focus<br>○ Gender (m/f): 5/6 | Riding a Pedalo for 7 meters | **Groups of interest:**<br>- External focus ('Implicit'): "Focus on pushing the platforms forward"<br>- Internal focus ('Explicit'): "Focus on pushing your feet forward"<br><br>**Procedure:**<br>*Day 1: Learning phase:*<br>- 20 ST trials at preferred pace<br>*Day 2: Test phase*<br>- 3 test blocks of 4 trials<br>○ ST (riding forward as fast as possible)<br>○ ST (riding backward as fast as possible)<br>○ DT (riding forward as fast as possible + counting aloud | ***Primary outcome:***<br>- Primary motor task: Movement time (s) (M±SD)<br>- Secondary task: Performance not assessed<br><br>***Secondary outcome:***<br>- Declarative knowledge: Not assessed | **Motor task performance:**<br>*Implicit*<br>- ST = 11.5<br>- DT = 15.8<br>- DTC= 37.4%<br>*Explicit*<br>- ST = 15.0<br>- DT = 19.1<br>- DTC= 27.3%<br><br>**Secondary task performance:** N/A<br>**Declarative knowledge:** N/A<br><br>**Implicit versus Explicit comparison:**<br>- Motor ST: *p*<0.001<br>- Motor DT: *p*<0.05<br>- Motor DTC: ? |

| | | | | | |
|---|---|---|---|---|---|
| | - External focus<br>o Gender (m/f): 5/6<br><br>**Pre-test single task motor performance:** N/A | | backward in threes from two-digit number) | | - Secondary DT: N/A<br>- Secondary DTC: N/A<br>- Declarative knowledge: N/A |
| Tse et al. (2017) [58] | **Number at baseline: 70**<br><br>**Inclusion/exclusion criteria:**<br>- No experience with task<br>- Right-handed<br>- No neurological disease<br>- No (chronic) pain in back and right forearm, shoulder or hand<br><br>**Number of groups:** 4<br>- Analogy – Young (n=18)<br>- Explicit – Young (n=18)<br>- Analogy – Old (n=17)<br>- Explicit – Old (n=17)<br><br>**Specific group characteristics:**<br>- Younger adults<br>o Gender (m/f): ?<br>o Age (years): 21.9±2.3<br>o MMSE: >24<br>o Digit span memory: 26±4.2<br>- Older adults<br>o Gender (m/f): ?<br>o Age (years): 66.9±4.6<br>o MMSE>24<br>o Digit span memory: 20.0±5.4<br><br>**Pre-test single task motor performance:** N/A | Table tennis task<br>- Hit table tennis ball onto target area with topspin using forehand stroke | **Groups of interest:**<br>- Analogy (‘Implicit’): “Move your racket such that it is travelling up the side of a mountain”<br>- Explicit (‘Explicit’): Received 9 basic instructions on how to hit topspin forehand<br><br>**Procedure:**<br>*Day 1: Learning phase:*<br>- 180 ST trials<br>*Day 1: Test phase 1*<br>- 2 test blocks of 30 trials<br>o DT (counting backwards)<br>o ST<br>*Day 2: Test phase 2*<br>- 1 test block of 30 trials<br>o ST | ***Primary outcome:***<br>- Primary motor task: Accuracy (number of points per block; M±SD)<br>- Secondary task: Performance not assessed<br><br>***Secondary outcome:***<br>- Declarative knowledge: Number of explicit rules (M± SD) | **Motor task performance:**<br>*Implicit-young*<br>- ST = 54.4±5.1<br>- DT = 44.5±5.3<br>- DTC = 18.3%<br>*Explicit-young*<br>- ST = 37.9±5.3<br>- DT = 26.6±5.7<br>- DTC = 30.0%<br>*Implicit-old*<br>- ST = 31.3±7.6<br>- DT = 25.0±7.3<br>- DTC = 20.3%<br>*Explicit-old*<br>- ST = 20.7±6.8<br>- DT = 15.9±7.3<br>- DTC = 23.4%<br><br>**Secondary task performance:** N/A<br><br>**Declarative knowledge:**<br>*Implicit-young:* 3.8±1.2<br>*Explicit-young:* 9.2±2.7<br>*Implicit-old:* 4.4±1.3<br>*Explicit-old:* 7.4±2.1<br><br>**Implicit versus Explicit comparison:**<br>*Young groups*<br>- Motor ST: *p*<.001<br>- Motor DT: *p*<.001<br>- Motor DTC: *p*>.05<br>- Secondary DT: N/A<br>- Secondary DTC: N/A<br>- Declarative knowledge: *p*<.001<br>*Old groups*<br>- Motor ST: *p*<.001<br>- Motor DT: *p*<.001 |

| | | | | | |
|---|---|---|---|---|---|
| | | | | | - Motor DTC: *p*>.05<br>- Secondary DT: N/A<br>- Secondary DTC: N/A<br>- Declarative knowledge: *p*<.001 |
| Wulf et al. (2001) [16] | **Number at baseline: 28**<br><br>**Inclusion/exclusion criteria:**<br>- No experience with task<br><br>**Number of groups:** 2<br>- Internal focus (n=14)<br>- External focus (n=14)<br><br>**General descriptives:**<br>- Gender (m/f): 5/23<br>- Age (years): ?<br><br>**Specific group characteristics:** N/A<br><br>**Pre-test single task motor performance:** N/A<br><br>**Pre-test single task cognitive performance:**<br>- Internal focus: 360±?<br>- External focus: 349±? | Balancing task - Keeping a 1-axial stabilometer horizontal | **Groups of interest:**<br>- External focus ('Implicit'): "Focus on markers attached to the platform, and keep them horizontal"<br>- Internal focus ('Explicit'): "Focus on feet, and keep them horizontal"<br><br>**Procedure:**<br>*Day 1+2: Learning phase:*<br>- 7 trials of 90-seconds<br>  o 6 DT (Probe reaction time task)<br>  o 1 ST<br>*Day 3: Test phase:*<br>- 7 trials of 90-seconds<br>  o 6 DT (Probe reaction time task)<br>  o 1 ST | ***Primary outcome:***<br>- Primary motor task: RMSE deviation from horizontal (M±SD)<br>- Secondary task: Probe reaction time (ms; M±SD)<br><br>***Secondary outcome:***<br>- Declarative knowledge: Not assessed | **Motor task performance:**<br>*Implicit*<br>- ST = 3.5<br>- DT = 3.3<br>- DTC = -5.7%<br>*Explicit*<br>- ST = 4.1<br>- DT = 4.2<br>- DTC = 2.4%<br><br>**Secondary task performance:**<br>*Implicit*<br>- ST = 295<br>- DT = 301<br>- DTC= 2.0%<br>*Explicit*<br>- ST = 307<br>- DT = 331<br>- DTC= 7.8%<br><br>**Declarative knowledge:** N/A<br><br>**Implicit versus Explicit comparison:**<br>- Motor ST: *p*<.05<br>- Motor DT: *p*<.05<br>- Motor DTC: *p*>.05<br>- Secondary DT: *p*<.01<br>- Secondary DTC: *p*<.001<br>- Declarative knowledge: N/A |

**NB:** P<0.05: Significant difference between implicit- and explicit group; ?: Outcome measure was assessed, but exact values could not be obtained. N/A: Outcome measure not applicable from report. Abbreviations: BBS=berg balance scale; DT= dual-task; DTC= dual-task costs; M=mean; MMSE=mini-mental state examination; PRT=probe reaction time; RMSE=root-mean-square error; SD=standard deviation; ST=single-task;
